# An Ultrasound-Responsive Bio-Adhesive Piezoelectric Hydrogel for Osteoarthritis Cartilage

**DOI:** 10.3390/gels12070630

**Published:** 2026-07-15

**Authors:** Yuan Li, Ziyu Chen, Shiyu Zhu, Yan Wei, Zhen Geng, Jianping Huang, Mengmeng Li

**Affiliations:** 1 Institute of Translational Medicine, Shanghai University, Shanghai 200444, China; 2 School of Medicine, Shanghai University, Shanghai 200444, China; 3 Sanming Institute of Translational Medicine, Sanming 365004, China

**Keywords:** injectable hydrogel, piezoelectric effect, bio-adhesive, chondrogenic differentiation, osteoarthritis treatment

## Abstract

Osteoarthritis (OA) is a degenerative joint disease characterized by progressive loss of articular cartilage and an associated decline in its intrinsic mechanoelectrical signaling. Current osteoarthritis treatments relieve symptoms but fail to prevent cartilage degeneration or restore its native biophysical microenvironment. Here, we present an ultrasound-activated, mussel-inspired bio-adhesive hydrogel that addresses these challenges by recreating the cartilage’s piezoelectric cues in situ while achieving stable intra-articular retention under synovial conditions. The hydrogel, denoted SFHD-BT@PDA, consists of a silk fibroin (SF) matrix integrated with dopamine-functionalized hyaluronic acid (HADA) and embedded barium titanate nanoparticles coated with polydopamine (BT@PDA). This multi-level design imparts strong interfacial adhesion to wet cartilage (via catechol-mediated bonding to collagen) and piezoelectric sensitivity to external ultrasound. Under ultrasound stimulation, SFHD-BT@PDA generates localized electrical microcurrents that recruit endogenous MSCs via electrotaxis and subsequently promote their chondrogenic differentiation. In vitro, ultrasound-triggered electrical cues upregulated chondrogenic markers (SOX9, collagen II, aggrecan) in MSCs and activated TGF-β signaling, demonstrating restoration of the pro-anabolic bioelectric microenvironment. In a murine DMM model, the adhesive hydrogel exhibited prolonged retention on cartilage surfaces and, with ultrasound, induced robust cartilage regeneration and OA reversal. Treated joints showed preserved proteoglycan and Type II collagen content, inhibited osteophyte formation, and protection of subchondral bone microarchitecture. In summary, this mussel-inspired piezoelectric hydrogel provides an electromechanical stimulation platform that effectively couples physical cues with bio-adhesion to regenerate cartilage.

## 1. Introduction

Osteoarthritis (OA) is a degenerative disease that mainly involves weight-bearing joints. Its incidence has risen sharply with the aging trend of the global population, making it the primary cause of motor function loss [1,2]. Clinically, intervention strategies for OA often fall into a dilemma: the early conservative treatment of drugs mainly relies on nonsteroidal anti-inflammatory drugs or corticosteroids, aiming to relieve pain symptoms, but it is difficult to curb the progressive degradation of the cartilage matrix [3]. When the disease progresses to the late stage, although joint replacement surgery can reconstruct the function, its invasive nature and limited implant lifespan remain unavoidable challenges [4,5,6,7]. Early OA is considered a critical therapeutic window [8]. However, current intervention strategies mostly focus on the accompanying biochemical factors, while neglecting the unique biophysical properties of cartilage and their core role in maintaining tissue homeostasis [2].

In fact, articular cartilage is not only a load-bearing mechanical cushion, but also a natural piezoelectric tissue [9,10]. The highly ordered collagen fiber bundles in the extracellular matrix (ECM) endow cartilage with the ability to transform physiological mechanical loads into endogenous microelectrical signals [11,12]. This subtle “mechanical–electrical coupling” mechanism not only helps to convert mechanical stress into electrical energy to minimize mechanical damage caused by adverse loads, but also constitutes a key physical clue for the regulation of chondrocyte anabolism and matrix remodeling [13]. However, during the pathological progression, with the collagen network destruction and the proteoglycan loss, this endogenous piezoelectric response gradually weakened or even silenced [14]. The loss of the bioelectric microenvironment not only weakens the synthetic stimulation to cells, but also further aggravates the metabolic disorder, thus forming irreversible pathological feedback [15]. Therefore, how to remodel the piezoelectric microenvironment of damaged cartilage in situ by exogenous means and restart the silent bioelectrical signal has become a key scientific problem for breaking the course of OA and inducing tissue regeneration.

Research has shown that local electrical stimulation can modulate intracellular gene transcription by interacting with ion channels and enzymes. This mechanism can not only promote the migration and proliferation of stem cells to the damaged area and their directional differentiation into chondrocytes, but also alleviate pain and accelerate the reabsorption of synovial effusions [16,17,18,19,20]. Lately, the application of piezoelectric biomaterials to respond to ultrasound in vitro to build a radio stimulation platform has emerged in tissue engineering owing to its non-invasive and spatio-temporal controllability [12,21,22]. Despite the great potential of piezoelectric nanomaterials such as barium titanate (BT) to induce stem cell differentiation in vitro, their translation into clinical intra-articular injection therapy still faces severe material challenges [23]. As a moist cavity with continuous dynamic friction and rich synovial fluid, the articular cavity has a very strong scavenging effect [24,25]. Due to the lack of an effective interface anchoring mechanism, traditional nanoparticles or ordinary hydrogel carriers are prone to migrate or metabolize rapidly under synovial fluid scouring, resulting in the retention time of drugs at the lesion site being much shorter than the physiological cycle required for cartilage repair [26]. This temporal mismatch between material retention and tissue regeneration fundamentally constrains the therapeutic efficacy of current piezoelectric strategies [27]. Recently, Lin et al. [28] reported an ultrasound-triggered PDA-modified BaTiO_3_-loaded HA hydrogel for OA treatment, demonstrating the promise of injectable piezoelectric hydrogels in inflammation modulation and chondrogenesis. However, stable wet adhesion to cartilage surfaces and long-term retention under synovial fluid scouring remain key challenges for intra-articular hydrogel therapy. Therefore, beyond constructing an ultrasound-responsive piezoelectric microenvironment, improving cartilage interfacial anchoring is also crucial for achieving sustained local therapeutic efficacy.

**Scheme 1:**
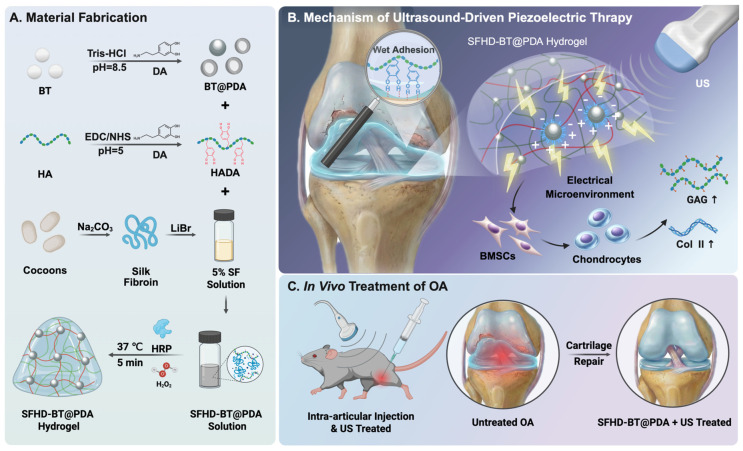
Schematic illustration of the fabrication, therapeutic mechanism, and in vivo application of SFHD-BT@PDA hydrogel. (**A**) Synthesis of BT@PDA nanoparticles and HADA precursors, and the formation process of the SFHD-BT@PDA hydrogel. (**B**) Therapeutic mechanism of the SFHD-BT@PDA hydrogel. (**C**) Procedure for OA treatment using SFHD-BT@PDA under US stimulation. Created in BioRender. Bigbone, B. (2026) https://BioRender.com/94ljuwr (accessed on 1 June 2026).

To address the above challenges, inspired by the strong wet adhesion of marine mussels in turbulent environments, we propose a systemic solution that combines “mechano-electrical response” with “bio-inspired adhesion” by constructing an injectable nanocomposite hydrogel system (SFHD-BT@PDA) [29,30,31]. In terms of material design, we did not simply perform physical blending but adopted a multi-level interface engineering strategy. First, polydopamine (PDA) was used for in situ surface modification of barium titanate nanoparticles to address the agglomeration of inorganic fillers while utilizing the abundant active sites of PDA to enhance its binding with the matrix [26]. Second, we introduced dopamine-functionalized hyaluronic acid (HADA) into the silk fibroin (SF) network. The molecular dynamics simulation provided molecular-level insight into the molecular basis of this design: the catechol group in HADA can form stable cation–π interactions and hydrogen bond networks with specific residues on the collagen surface, thereby endowing the hydrogel with excellent adhesion capabilities on wet cartilage surfaces [32,33]. This study seeks to demonstrate that the molecularly engineered SFHD-BT@PDA hydrogel serves a dual purpose: it acts as a physical matrix hosting piezoelectric efficiency particles while, more importantly, achieving long-term retention within the joint cavity by virtue of its robust interfacial integration capabilities. Under the stimulation of LIPUS in vitro, the bionic piezoelectric microenvironment constructed by this system can efficiently recruit endogenous bone marrow mesenchymal stem cells (BMSCs) through electrical tropism, and activate key signaling pathways such as TGF-β to induce them to differentiate into the cartilage lineage. Through this “retention–recruitment–differentiation” cascade effect, we hope to provide an innovative strategy with clinical transformation potential for the reversal treatment of early OA (Scheme 1).

## 2. Results and Discussion

### 2.1. Construction and Characterization of BT@PDA Nanoparticles

**Figure 1:**
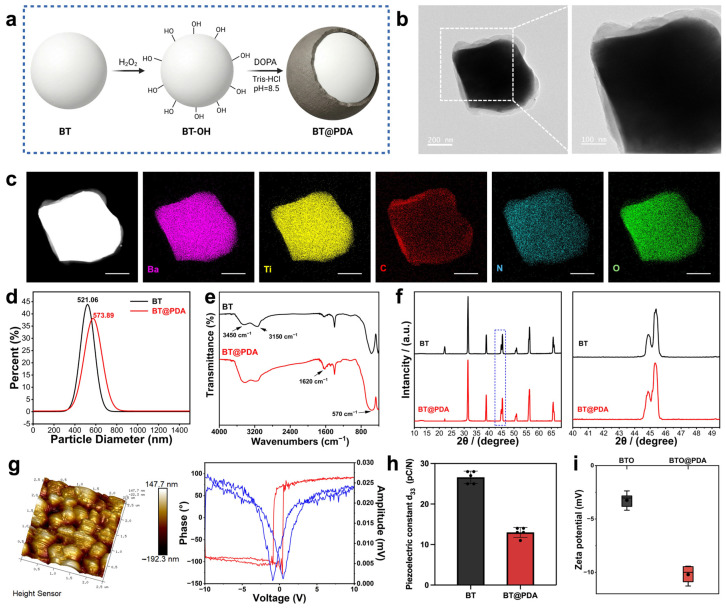
Synthesis and characterization of BT@PDA nanoparticles. (**a**) Schematic diagram of the preparation of BT@PDA nanoparticles via in situ dopamine polymerization. Created in BioRender. Bigbone, B. (2026) https://BioRender.com/xo5d68d (accessed on 1 June 2026). (**b**) TEM images of BT@PDA with different magnifications. (**c**) STEM image and corresponding EDS elemental mapping of Ba, Ti, C, N, and O. (Scale bar = 200 nm). (**d**) Particle size distribution of BT and BT@PDA measured by DLS. (**e**) FTIR spectra of BT and BT@PDA. (**f**) XRD patterns of BT and BT@PDA. The right panel presents an enlarged view of the diffraction peaks highlighted by the blue dashed box in the left panel. (**g**) PFM amplitude butterfly curves and phase hysteresis loops of BT@PDA. (**h**) Quantitative piezoelectric constant (*d*_33_) of BT and BT@PDA (*n* = 5). (**i**) Zeta potential measurements of BT and BT@PDA nanoparticles (*n* = 5).

In this work, BT nanoparticles were selected as the core functional unit for constructing the piezoelectric microenvironment. The provision of a robust piezoelectric microenvironment largely depends on the successful construction of a homogeneous piezoelectric hydrogel. However, due to their high surface energy, raw BT nanoparticles are prone to severe agglomeration in aqueous matrices, which hinders the internal crosslinking of the SF hydrogel during gelation and makes it difficult to form a uniform piezoelectric microenvironment. To address this, we introduced PDA as a biomimetic surface modifier. Utilizing the abundant active catechol and quinone imine groups in PDA, a coating layer was formed via in situ self-polymerization of dopamine on the hydroxylated BT surface (Figure 1a). This strategy endowed the nanoparticles with a hydrophilic phenolic hydroxyl surface, which not only effectively screened the high surface energy attraction between particles but also provided abundant active sites for binding with the hydrogel matrix, thereby significantly enhancing the compatibility at the interface between the inorganic filler and the biopolymer network.

To verify the successful construction of the core–shell structure, particle morphology was characterized using TEM and HAADF-STEM. Morphological and elemental comparisons further confirmed the successful PDA coating. As shown in Supplementary Figure S1c, the uncoated bare BT nanoparticles exhibited a sharp interfacial boundary, and their EDS elemental mapping showed the expected Ba, Ti, and O distributions. In contrast, the BT@PDA nanoparticles displayed a distinct core–shell structure, together with newly detected C and N signals uniformly distributed across the particles (Figure 1b,c), indicating the successful formation of the PDA coating layer. The high-contrast crystalline BT core was uniformly coated by a low-contrast amorphous PDA layer with a thickness of approximately 25–30 nm. DLS analysis showed that the diameter of BT@PDA increased compared to the original BT (Figure 1d), which further confirmed the existence of surface coating. EDS elemental mapping map (Figure 1c) confirmed the spatial distribution of elements, showing that Ba and Ti signals were mainly concentrated in the core region, while C, N, and O signals covered the entire particle. Notably, the N element signal specific to dopamine residues highly coincides with the particle contour, which strongly proves the integrity and continuity of the PDA coating.

The chemical structure of BT@PDA was analyzed by FTIR. As shown in Figure 1e, compared with the original BT, the spectrum of BT@PDA shows new characteristic absorption peaks at 1620 cm^−1^ (N–H bending vibration) and broad absorption at 3150–3450 cm^−1^ (phenolic hydroxyl –OH/N–H stretching vibration), confirming successful deposition of PDA. The crystal structure analysis (XRD, Figure 1f) for both BT and BT@PDA particles showed that the signature split diffraction peaks were observed at 2θ = 45°, indicating that the main tetragonal perovskite structure was retained after PDA modification. Surface hydroxylation and PDA encapsulation induce minor changes in peak intensity and profile at 22°, 45°, and 56°, which stem from interfacial disturbance and outer-layer mechanical restriction instead of new crystal phases [34,35]. No impurity phase or peak position shift was detected in the spectrum, indicating that mild polymerization conditions did not damage the crystallinity of the ferroelectric core, which laid a structural foundation for its piezoelectric properties.

Furthermore, we used piezoelectric force microscopy (PFM) to evaluate the electromechanical coupling properties of the materials to confirm their applicability in cartilage tissue engineering. BT@PDA exhibited the canonical butterfly amplitude loops and 180° phase hysteresis (Figure 1g), confirming ferroelectric domain switching and retained piezoelectricity. Quantitative analysis (Figure 1h) shows that an effective piezoelectric coefficient (*d*_33_) of BT@PDA is about 13 pC/N, lower than that of the original BT (about 26 pC/N). The degradation of piezoelectric properties is primarily attributed to the dielectric shielding effect and mechanical clamping effect produced by the non-piezoelectric polymer shell. However, in the context of cartilage regeneration, this moderate piezoelectric stimulation, by simulating the microelectric environment of natural cartilage, is more inclined to trigger stem cell differentiation into chondrocytes, avoiding the osteogenic differentiation tendency and cell damage that may be caused by an excessively high-voltage electrical signal [36].

In addition, Zeta potential test results (Figure 1i) showed that the particle surface potential decreased from −4.3 mV for BT to −10.1 mV for BT@PDA. The increase in surface negative-charge density significantly enhanced the electrostatic repulsion between particles, effectively counteracting van der Waals forces, thereby greatly enhancing nanoparticle dispersion stability in the aqueous system.

### 2.2. Preparation and Characterization of the SFHD-BT@PDA Hydrogel System

Engineering a hydrogel structure that synergizes mechanical robustness with seamless interfacial integration is the cornerstone for reconstructing a functional bioelectric microenvironment. Silk fibroin was selected as the backbone material to mimic the collagen fibril network of natural cartilage due to its superior mechanical properties and tunable biodegradability. However, pure SF hydrogels usually lack sufficient wet tissue adhesiveness, making it difficult to form a tight fit with host cartilage tissue after injection, which easily leads to graft detachment or integration failure.

**Figure 2:**
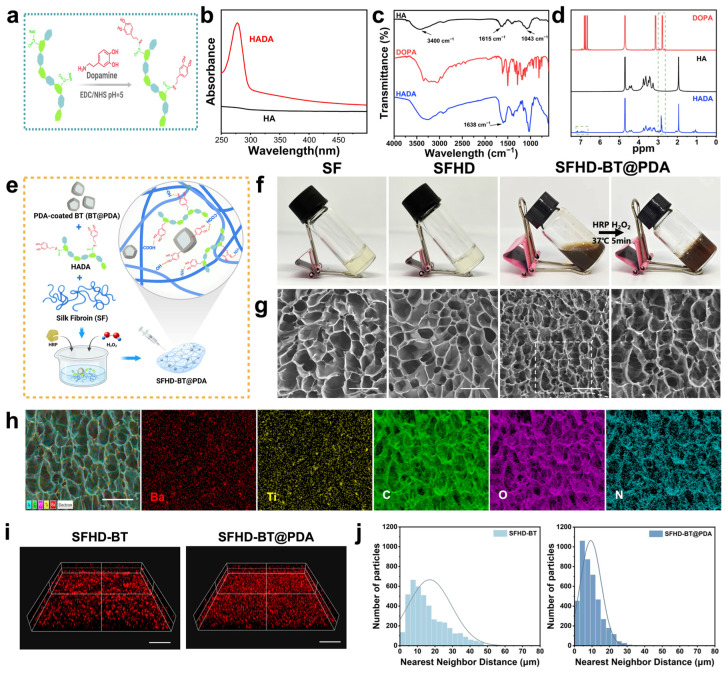
Preparation and microstructural characterization of the SFHD-BT@PDA hydrogels. (**a**) Synthetic route of HADA. Created in BioRender. Bigbone, B. (2026) https://BioRender.com/kpurp4g (accessed on 1 June 2026). (**b**) UV-vis absorption spectra of HA and HADA. (**c**) FTIR spectra of HA, DOPA, and HADA. (**d**) ^1^H-NMR spectrum of HADA. (**e**) Schematic diagram of the preparation process for the SFHD-BT@PDA composite hydrogel. Created in BioRender. Bigbone, B. (2026) https://BioRender.com/m7mohca (accessed on 1 June 2026). (**f**) Photographs of the hydrogel sol–gel transition. (**g**) SEM images of SF, SFHD, and SFHD-BT@PDA hydrogels (Scale bar = 50 μm). (**h**) EDS elemental mapping of the SFHD-BT@PDA hydrogel (Scale bar = 50 μm). (**i**) 3D confocal fluorescence reconstructions showing the spatial distribution of nanoparticles in the SFHD-BT and SFHD-BT@PDA hydrogels (Scale bar = 100 μm). (**j**) Quantitative histograms of the Nearest Neighbor Distance (NND) corresponding to the nanoparticle distributions in panel I.

To overcome the above limitations, HADA was first synthesized by EDC/NHS mediated amide condensation reaction and introduced into the SF matrix as a bioactive adhesion component (Figure 2a). The design of HADA follows a dual functional rationale: biologically, the natural ligand affinity of HA for the CD44 receptor on the surface of BMSCs is used to initiate the chondrogenic differentiation signaling pathway; chemically, the grafted catechol groups are used as “mussel biomimetic” anchors, which not only provide wet tissue adhesion, but also serve as highly active sites for horseradish peroxidase (HRP) catalyzed crosslinking, thereby accelerating the in situ gelation process of SF based hydrogels.

The chemical structure of HADA was confirmed by multidimensional spectral analysis. UV-vis spectra (Figure 2b) showed that HADA exhibited a visible characteristic absorption peak at 280 nm compared with the original HA, which was attributed to the benzene ring structure of the catechol group. FTIR spectra (Figure 2c) further confirmed the occurrence of the grafting reaction, and the nascent absorption bands corresponding to amide bonds (Amide I and II) and aromatic ring skeletal vibrations. ^1^H-NMR spectrum (Figure 2d) revealed that the characteristic resonance signal of aromatic protons of dopamine residues was detected everywhere at δ6.7–6.9 ppm. These data collectively confirmed the successful covalent modification of dopamine side groups on the HA backbone, laying a material foundation for subsequent enzymatic crosslinking and wet-state adhesion.

Building on this, we introduced BT@PDA nanoparticles with a core–shell structure into SF/HADA precursor solution, and used HRP/H_2_O_2_ to trigger oxidative coupling to prepare a composite hydrogel system (Figure 2e). The sol–gel transition behavior was assessed using the inverted tube method (Figure 2f). The results showed that the precursor solution completed the sol–gel transition within 5 min at 37 °C, the physiological temperature. This dynamic window of rapid gelation is conducive to clinical minimally invasive injection operation, which can not only ensure the fluidity during injection, but also prevent the loss of hydrogel after injection.

Microstructural analysis via scanning electron microscopy (SEM) (Figure 2g) demonstrated that the hydrogels possessed a highly connected porous structure, which constructs a hierarchical nanotopography favorable for cell attachment and spreading. The uniform spatial distribution of the functional components was verified by EDS elemental mapping (Figure 2h), which showed highly overlapping signals of Ba, Ti, C, O, and N. Crucially, the PDA coating played a pivotal role in maintaining structural integrity by preventing the agglomeration of the high-surface-energy inorganic fillers. Comparative EDS mapping (Figure S3) revealed severe aggregation of Ba and Ti signals in the unmodified SF-BT hydrogel. In contrast, the SF-BT@PDA hydrogel exhibited a highly uniform Ba and Ti distribution across the network. 3D confocal laser scanning microscopy (Figure 2i) and corresponding Nearest Neighbor Distance (NND) quantitative analysis (Figure 2j) were utilized to evaluate nanoparticle dispersion. While raw BT nanoparticles in the unmodified SFHD-BT group exhibited severe macroscopic aggregation, the BT@PDA nanoparticles were uniformly dispersed throughout the polymer matrix. The NND histogram for SFHD-BT@PDA showed a significantly narrower and more homogeneous interparticle distance distribution, confirming that the catechol-rich PDA shell effectively mitigated nanoparticle aggregation via multiple hydrogen bonds and π-π stacking with the hydrogel network.

**Figure 3:**
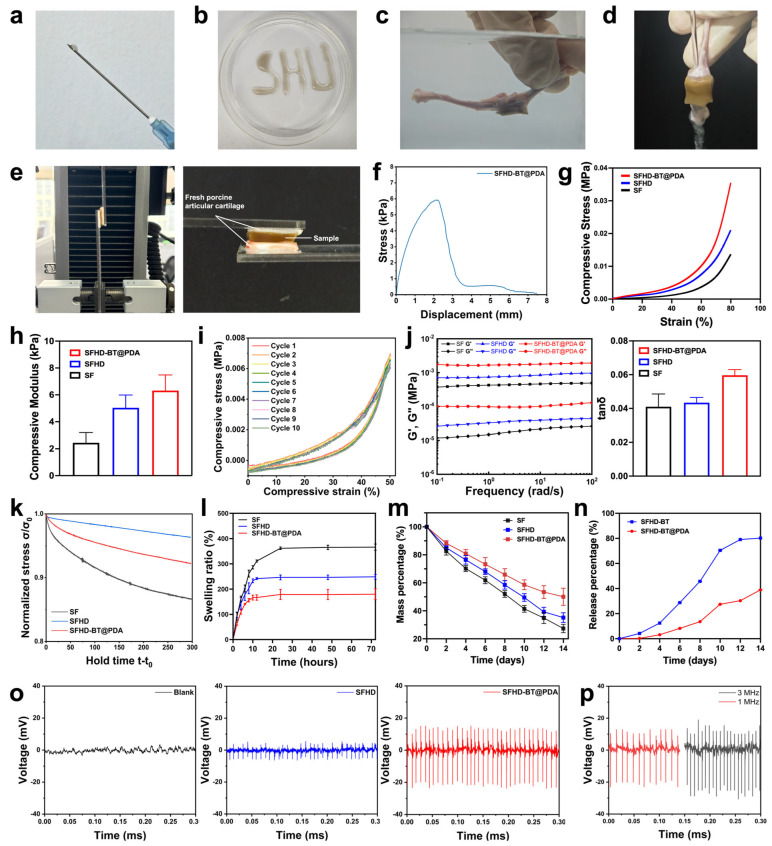
Mechanical, adhesive, and piezoelectric characterizations of the SFHD-BT@PDA hydrogel. (**a**,**b**) Injectability demonstration: The hydrogel can be extruded through a 26G needle (**a**) and written into specific letters (**b**). (**c**,**d**) Adhesion assessment on fresh rat articular cartilage, demonstrating stable anchorage under (**c**) static immersion and (**d**) dynamic water flushing. (**e**,**f**) Lap-shear test: (**e**) Experimental setup using fresh porcine articular cartilage and (**f**) representative stress–displacement curve. (**g**,**h**) Compressive mechanical performance: (**g**) Representative compressive stress–strain curves and (**h**) calculated compressive modulus for each group (*n* = 3). (**i**) Cyclic compressive stress–strain hysteresis loops of the SFHD-BT@PDA hydrogel under 50% strain for 10 cycles. (**j**) Rheological frequency sweep measurements, showing *G′*, *G″* (**left panel**), and *tan δ* values (**right panel**) for the hydrogels. (**k**) Normalized stress–relaxation profiles over time. (**l**) Swelling ratio, (**m**) in vitro degradation, and (**n**) release kinetics profiles of the hydrogels (*n* = 3). (**o**) Real-time voltage output of different hydrogel groups: Blank: SFHD-BT@PDA hydrogel without ultrasound stimulation; SFHD: SFHD hydrogel with ultrasound stimulation; SFHD-BT@PDA: SFHD-BT@PDA hydrogel with ultrasound stimulation. (**p**) Voltage output comparison of the SFHD-BT@PDA hydrogel under 1 MHz versus 3 MHz ultrasound.

For minimally invasive applications, hydrogels need to have good injectability and in situ retention. Hydrogels should possess suitable injectability before complete gelation and stable retention after in situ network formation. The SFHD-BT@PDA precursor solution remained sufficiently flowable during the HRP/H_2_O_2_-triggered sol–gel transition window, allowing it to be smoothly extruded through a 26 G needle and written into a predefined pattern (Figure 3a,b), indicating its excellent injectability. In addition, due to the strong adhesiveness of catechol groups, SFHD-BT@PDA showed excellent wet tissue adhesion. In order to simulate the physiological challenge of an articular fluid-rich environment on tissue adhesion (Figure 3c,d), we adhered SFHD-BT@PDA hydrogel to the surface of fresh rat knee cartilage and immersed it in PBS. It was observed that the hydrogel not only maintained stable adhesion during static immersion (Figure 3c), but also did not peel or shift under severe dynamic water flow scouring (Figure 3d). This wet anchoring ability has important clinical significance for preventing the displacement of hydrogel caused by joint fluid flow. To quantitatively assess this wet tissue adhesiveness, lap-shear tests using fresh porcine articular cartilage were conducted (Figure 3e). The corresponding stress–displacement curve (Figure 3f) revealed a peak interfacial adhesive strength of approximately 5.9 kPa, confirming the reliable anchoring capability provided by the catechol groups in the HADA component.

Compressive stress–strain analysis (Figure 3g,h) demonstrated that the incorporation of BT@PDA nanoparticles enhanced the mechanical rigidity of the composite, increasing the compressive modulus to approximately 6.2 kPa compared to 2.4 kPa for the pure SF matrix. Moreover, 10-cycle loading tests at 50% strain (Figure 3i) revealed wonderful dynamic fatigue resistance and shape recoverability. Quantitative analysis revealed a stable hysteresis energy of approximately 0.293 kJ/m^3^ and a consistent energy dissipation ratio of approximately 20% throughout the cycles. This reliable viscoelastic damping performance confirms the hydrogel’s ability to efficiently dissipate external mechanical stress, biomimetically replicating the shock-absorbing function of natural articular cartilage under dynamic physiological loads.

The rheological and compressive mechanical characterizations demonstrate consistent reinforcement trends across the hydrogel groups. Rheological frequency sweeps (Figure 3j) confirmed a stable viscoelastic profile, with the storage modulus (*G′*) remaining consistently higher than the loss modulus (*G″*) across the tested range. To further evaluate the viscoelasticity of the hydrogels, the loss tangent (*tan δ = G″/G′*) was analyzed. All hydrogels exhibited *tan δ* values well below 1, confirming their solid-like elastic networks. Notably, the SFHD-BT@PDA composite hydrogel demonstrated a concurrent increase in both the storage modulus (*G′*) and the *tan δ* value. This indicates that the introduction of BT@PDA endows the composite hydrogel with excellent shock-absorbing capacity alongside enhanced stiffness. The normalized stress–relaxation profiles (Figure 3k) indicated a viscoelastic energy-dissipation behavior that effectively mimics the natural cartilage extracellular matrix.

Finally, the physicochemical stability of the system was assessed. The SFHD-BT@PDA hydrogel exhibited an anti-swelling property, stabilizing at a relatively low equilibrium swelling ratio of roughly 180%, compared to over 360% for the pure SF hydrogel (Figure 3l). This prevents potential tissue compression caused by excessive volume expansion in vivo. In vitro degradation assays (Figure 3m) demonstrated that the composite structure significantly delayed mass loss, retaining 50.06% of its mass at day 14, whereas the pure SF group and SFHD group degraded to below 40%. Concurrently, the cumulative release profile (Figure 3n) showed that the PDA coating facilitated a sustained release behavior, restricting the 14-day nanoparticle release percentage to 38.91%. These characteristics collectively ensure long-term structural integrity and sustained therapeutic efficacy within the joint cavity.

To verify the construction of the bioelectric microenvironment, the real-time piezoelectric output was recorded under ultrasound stimulation (Figure 3o,p). The Blank group exhibited negligible electrical responses. The pure SFHD group showed a subtle and trace electrical response, which is attributed to the inherent weak piezoelectricity of the SF matrix. In contrast, the SFHD-BT@PDA hydrogel exhibited a stable, periodic, and significantly higher piezoelectric voltage with an amplitude of approximately 20 mV. This confirmed that while the SF matrix provides a baseline bioelectric hint, the embedded BT@PDA nanoparticles are the primary contributors to the efficient mechano-electrical coupling of the system. Additionally, the voltage output demonstrated a frequency-dependent response when comparing 1 MHz and 3 MHz stimulations (Figure 3p).

### 2.3. Molecular Dynamics Simulation of HADA–Collagen Interfacial Adhesion

While macroscopic assays have validated the robust integration of SFHD-BT@PDA hydrogels with cartilage tissue, elucidating the microscopic binding mechanism between the adhesive HADA moiety and Type II collagen, the primary matrix component, is critical. So we engineered a molecular docking model pairing the HADA monomer with the collagen triple helix. Using MD and mm/GBSA calculation methods, analyze the atomic-level interaction patterns and thermal stability [33]. Constrained by computational limits, the HADA monomer was utilized as a simplified model. Although it cannot fully capture the conformational complexity and multivalent synergistic effects of polymer networks, it effectively reveals the fundamental local interaction motifs driving wet adhesion at the interface, rather than predicting the macroscopic behavior of the entire hydrogel.

**Figure 4:**
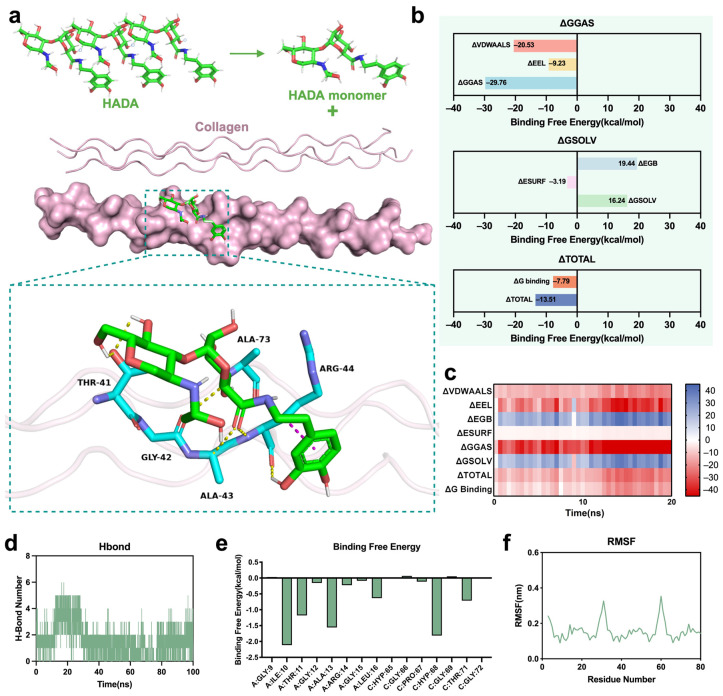
MD simulations of the HADA–collagen interface. (**a**) Molecular docking conformation of HADA with the collagen triple helix. The enlarged view (**bottom**) shows specific interactions, including hydrogen bonds (yellow dashed lines) and cation–π interactions (pink dashed lines). (**b**) Binding free energy ($∆G_{binding}$) and its energetic components. (**c**) Time-evolution heatmap of energy contributions (20 ns). (**d**) Dynamic fluctuation of intermolecular hydrogen bonds (100 ns). (**e**) Residue-based binding free energy calculation results. (**f**) RMSF profiles of residues at the binding interface.

The results of molecular docking intuitively show the binding conformation of HADA on the collagen surface (Figure 4a). HADA does not simply adsorb on the collagen surface, but adjusts its spatial conformation and accurately embeds into the groove of the collagen helix, with high geometric complementarity. The enlarged interaction diagram reveals a dense network of intermolecular forces. The skeleton of HADA and specific collagen residues (such as THR-41, etc.) form multiple hydrogen bonds (shown by the yellow dashed lines in the figure). The electron-rich benzene ring of the dopamine side chain forms an obvious cation–π interaction with the positively charged guanidine group of collagen ARG-44 (shown by the pink dashed lines in the figure). This non-covalent interaction that is still strong under water is considered to be the core source of the mussel-inspired wet adhesion mechanism.

To quantify the thermodynamic characteristics of this binding process, we calculated the binding free energy ($∆G_{binding}$) of HADA with collagen (Figure 4b). The binding free energy ($∆G_{binding}$) is −7.79 kcal/mol, which indicates that the binding of HADA to collagen is thermodynamically spontaneous. The energy decomposition analysis reveals that the adhesion is driven by van der Waals forces ($∆E_{vdW}$ = −20.53 kcal/mol) and electrostatic interactions ($∆E_{eel}$ = −9.23 kcal/mol). Van der Waals force is dominant in the bonding process, which indicates that the flexible chain of hada and the rigid helix of collagen achieve close molecular packing and full atomic contact; Electrostatic interaction mainly comes from the specific attraction between polar groups on both sides.

In the 100 ns simulation, the dynamic stability of the HADA–collagen complex was evaluated. In the heat map of binding energy versus time (Figure 4c), all items in all simulations were negative (corresponding to red), and the complex was stable without dissociation. In the hydrogen bond analysis (Figure 4d), intermolecular hydrogen bonds had no fluctuation from 1 to 6 ns, with an average of about 2–3. “Breakage-rearrangement” dynamic equilibrium is a typical feature of the adhesion of tough hydrogels, enabling the interface to effectively dissipate energy during continuous breakage. RMSF analysis (Figure 4f) shows that the fluctuation amplitude of the binding site residues is low (less than 0.4 nm), which indicates that the HADA molecule firmly anchors the collagen skeleton and restricts the local movement of the corresponding fragments. During the simulation, the radius of gyration (Rg, Figure S5a) characterizing the compactness of the macromolecular structure was about 2.50~2.55 nm. The results showed that the complex maintained a compact spherical or cylindrical conformation in the dynamic water environment, and neither looseness nor swelling occurred, thus maintaining the compactness of the bonding interface. The analysis results of solvent accessible surface area (SASA, Figure S5b) showed that the total surface area of the complex fluctuated between 60 and 64 nm^2^ without drastic changes. The stability of SASA reflects the complete embedding of the hydrophobic core and the stable contact of the ligand–receptor interface, indicating that water molecules will not destroy the binding interface between HADA and collagen, indicating that the complex system can bind stably in wet environment.

We analyze the energy at the amino acid residue level to find the hotspots of adhesion interface binding (Figure 4e). Specific residues of the collagen chain, such as ILE-10, ALA-13, and GLY-68, have obvious negative binding free energy (<−1.0 kcal/mol), and become the main molecular anchoring sites. The Root Mean Square Deviation (RMSD, Figure S5c) curve showed that the backbone atoms of the complex reached equilibrium rapidly at the beginning of the simulation and maintained a low fluctuation range of 0.2–0.3 nm throughout the simulation period. This stable RMSD trajectory without significant drift indicates that the introduction of HADA did not cause distortion of the collagen triple helix structure, and the complex system had reached a stable thermodynamic equilibrium state.

### 2.4. In Vitro Biocompatibility of SFHD-BT@PDA Hydrogels Under Ultrasound

**Figure 5:**
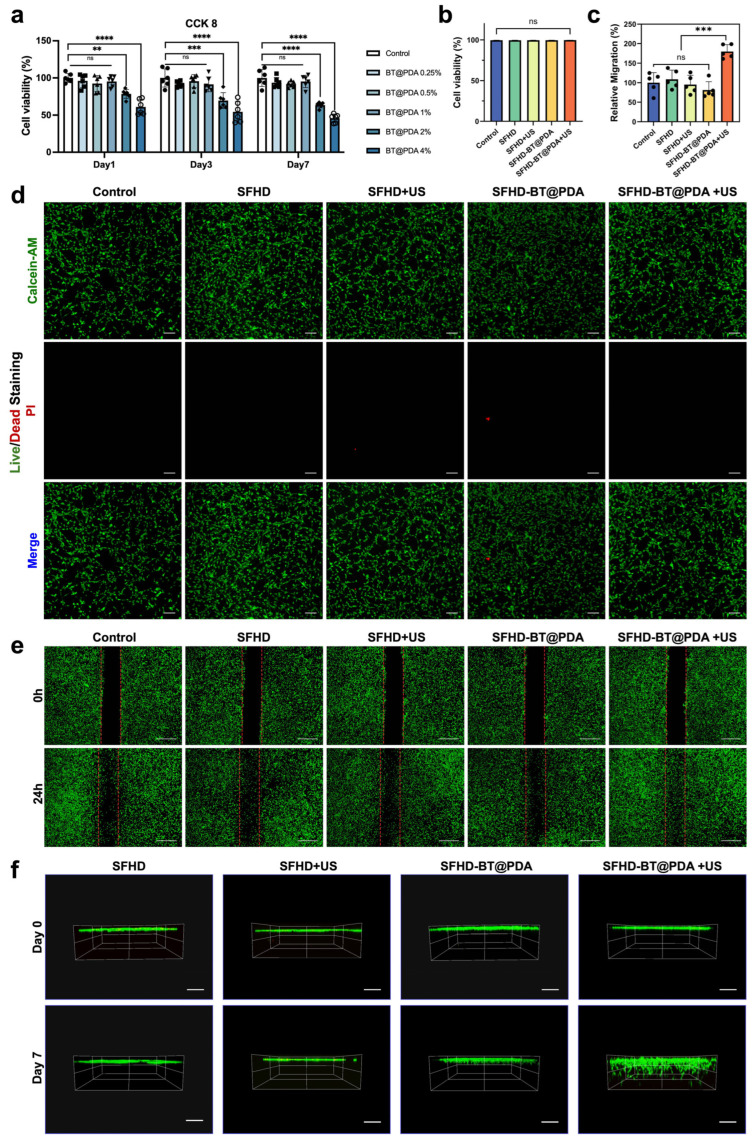
In vitro biocompatibility, proliferation, and migration evaluation of BMSCs. (**a**) Analysis of CCK-8 assays, the viability of BMSCs cultured on hydrogels with different BT@PDA concentrations (0.25–4%) under ultrasound stimulation on days 1, 3, and 7 (*n* = 6). (**b**) Quantitative analysis of cell viability based on live/dead staining (*n* = 5). (**c**) Quantitative analysis of the relative migration rate of BMSCs in each group (*n* = 5). (**d**) Live/dead staining images of BMSCs after 3 days of culture (Green: live cells; Red: dead cells; Scale bar = 100 μm). (**e**) Fluorescence images of the scratch assay at 0 h and 24 h (Scale bar = 500 μm). (**f**) 3D confocal reconstruction (Z-stack) showing the vertical infiltration of BMSCs into the hydrogel on days 0 and 7 (Scale bar = 200 μm). Data are presented as mean ± SD (** *p* < 0.01, *** *p* < 0.001, **** *p* < 0.0001, ns: no significance).

Ensuring the biosafety of nanocomposite hydrogels under specific treatment regimens is a prerequisite for their clinical application. Unlike conventional static toxicity tests, this study evaluated the long-term proliferation of BMSCs under periodic ultrasound stimulation (every other day) to simulate real in vivo treatment scenarios. To identify the optimal concentration of BT@PDA nanoparticles within the hydrogel system, we co-cultured BMSCs with hydrogels containing different concentrations of BT@PDA (0.25–4%) and applied ultrasound stimulation every other day. As shown in Figure 5a, cell viability exhibited a clear concentration-dependent response to the piezoelectric microenvironment. Under low concentrations (0.25%, 0.5%, 1%), the metabolic activity of BMSCs after 7 days of culture is relatively high (>90%), similar to that of the control group. This indicates that even in the presence of cyclic piezo stimulation, the cell compatibility of 1% BT@PDA hydrogel is also good. When the concentration reaches 2% and 4%, the cell viability is significantly reduced. High-load cell toxicity may be due to “over-stimulation”. Excessive piezoelectric voltage is generated when high-density nanoparticles are subjected to ultrasound, which exceeds the tolerance of cells. Therefore, 1% is determined as the optimal safe value for the concentration of BT@PDA and is applied to all subsequent experiments.

To observe the situation of cells under dynamic stimulation, the optimized SFHD-BT@PDA (1%) group was subjected to live/dead staining (Figure 5d) and quantitative analysis (Figure 5b) to determine whether this scheme is safe. In the image, the density of highly live cells (green) is relatively high, the number of dead cells (red) is very small, and the cell viability exceeds 95%. This also indicates that the SFHD-BT@PDA hydrogel with a concentration of 1% BT@PDA still maintains good biocompatibility under ultrasound.

### 2.5. Ultrasound-Stimulated SFHD-BT@PDA Hydrogel Promotes In Vitro BMSC Migration

Effective recruitment of endogenous stem cells to inflammatory sites is crucial for cartilage regeneration in osteoarthritis. To evaluate the ability of the piezoelectric hydrogel to mobilize bone marrow mesenchymal stem cells, we carried out a hydrogel-covered scratch experiment to simulate the migration of host cells toward the ultrasound-stimulated sites. Fluorescence imaging results showed (Figure 5e) that uniform scratch gaps with the same width were formed in all experimental groups at 0 h; after 24 h of culture, the migration behavior of cells in each group showed significant differences. Compared to the control, SFHD, SFHD + US, and SFHD-BT@PDA groups, the migration ability of BMSCs in the SFHD-BT@PDA + US group was significantly improved. Quantitative analysis based on the number of migrated cells in the scratch area (Figure 5c) confirmed this observation. The relative mobility of the SFHD-BT@PDA + US group was about 1.8 times that of the control group. This phenomenon proves that under ultrasonic excitation, the built-in dynamic electric field generated by the sb hydrogel provides directional guidance for BMSCs and drives cells to migrate efficiently.

To study the deep recruitment of cells in the 3D microenvironment, we reconstructed the 3D spatial distribution of BMSCs in the hydrogel within 7 days using a confocal laser scanning microscope (Figure 5f). Furthermore, the 3D cell infiltration capability within the hydrogel matrices was evaluated via Z-stack confocal imaging. As shown in Figure 5f, immediately after seeding (Day 0), BMSCs were uniformly distributed on the top surfaces across all groups. Interestingly, following 7 days of culture, the SFHD-BT@PDA + US group exhibited profound vertical cell migration, with cells penetrating deeply into the 3D porous network of the hydrogel. In contrast, cells in the other groups remained predominantly confined to the superficial layer. This result shows that the piezoelectric hydrogel not only helps the attachment of cells on the surface but also provides a continuous electrical signal guidance to help stem cells migrate to the deep layer and integrated with hydrogel in the three-dimensional space. This excellent full-layer colonization ability indicates that the piezoelectric hydrogel can interact with substances on the cell surface and provide continuous deep bioelectric guidance for migration to ensure the three-dimensional integration of stem cells and the hydrogel.

### 2.6. Ultrasound-Stimulated SFHD-BT@PDA Hydrogel Promotes Chondrogenic Differentiation of BMSCs

The therapeutic efficacy of injectable hydrogels for OA mainly depends on their ability to provide a pro-chondrogenic microenvironment, thereby inducing stem cell differentiation into chondrocytes and promoting matrix regeneration within the joint cavity. To evaluate the potential of the piezoelectric microenvironment in regulating stem cell fate, we investigated the chondrogenic differentiation behavior of BMSCs on different hydrogels with or without ultrasound stimulation.

**Figure 6:**
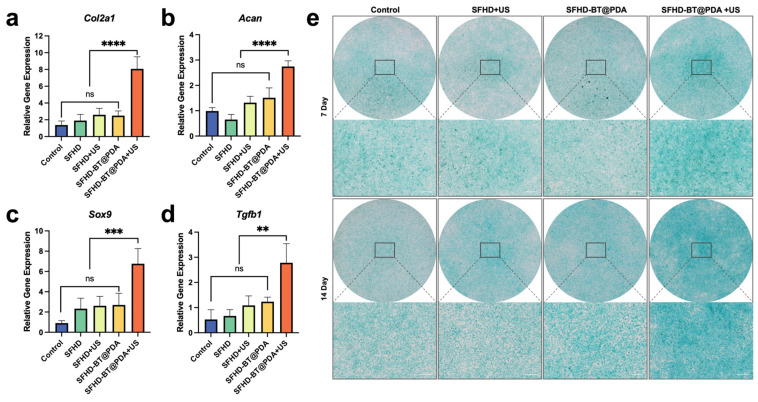
Assessment of ultrasound-mediated chondrogenic differentiation in vitro. ((**a**–**d**,) ** *p* < 0.01, *** *p* < 0.001, **** *p* < 0.0001, ns non-significant.) Transcriptional profiling of chondrogenic markers via RT-qPCR at day 14: (**a**) *Col2a1*, (**b**) *Acan*, (**c**) *Sox9*, and (**d**) *Tgfb1*. (**e**) Visualization of glycosaminoglycan (GAG) deposition via Alcian Blue staining at days 7 and 14 (Scale bar = 200 μm).

We first assessed the expression of chondrogenesis-specific genes in BMSCs at the mRNA level via RT-qPCR (Figure 6a–d). The results showed that, compared to all other groups, the SFHD-BT@PDA + US group induced a significant upregulation of key chondrogenic markers. Specifically, the expression of *Sox9*, the early master transcription factor, was markedly elevated, inducing the significant upregulation of extracellular matrix genes *Col2a1* and *Acan*. At the same time, the increase in *Tgfb1* level indicates that the electrical signal generated by the “ultrasonic–piezoelectric conversion” successfully initiates the TGF-β signaling pathway cascade, which is essential for maintaining the chondrogenic phenotype. However, such lineage-specific differentiation cannot be effectively induced by the mechanical effect of ultrasound alone.

Furthermore, we used Alcian Blue staining to observe the deposition of glycosaminoglycans (GAGs) in the ECM of BMSCs (Figure 6e). The staining results showed that the positive signal was significantly enhanced with the introduction of piezoelectric stimulation. The SFHD-BT@PDA + US group exhibited the most obvious deep-blue positive signals, forming dense cartilage-like nodules. Consistent with the macroscopic staining observations, the quantitative analysis of eluted Alcian Blue dye (Figure S6) further confirmed that the SFHD-BT@PDA + US group yielded the highest GAG accumulation at both day 7 and day 14 (*p* < 0.01 compared to the control group). This result further corroborated the gene expression data and proved that piezoelectric stimulation provided a sustained driving force for the synthesis and accumulation of the hyaline cartilage matrix.

**Figure 7:**
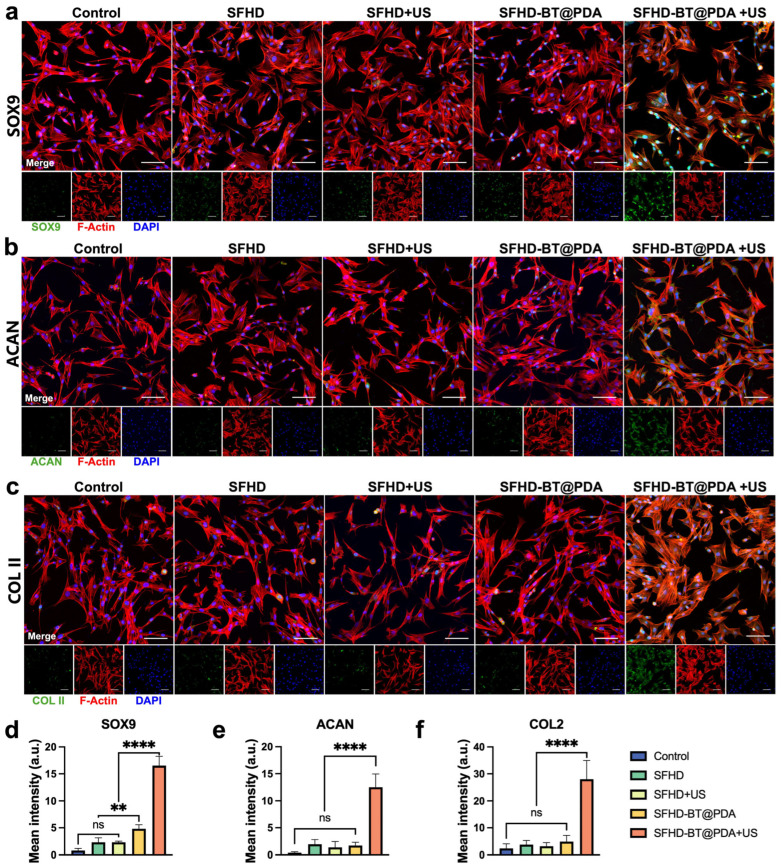
Immunofluorescence evaluation of ultrasound-mediated chondrogenic differentiation of BMSCs in vitro. (**a**–**c**) Representative immunofluorescence micrographs of chondrogenic proteins at day 14: (**a**) SOX9, (**b**) Aggrecan (ACAN), and (**c**) Type II Collagen (COL2) (Scale bar = 50 μm). The color variations in the merged fluorescence images mainly reflect differences in the localization and overlap of the three channels. SOX9, a nuclear transcription factor, partially overlaps with DAPI staining, producing a blue–green nuclear signal. In contrast, ACAN and COL2 are cartilage matrix-associated proteins mainly detected in cytoplasmic/pericellular or matrix regions, resulting in different overlap patterns with F-actin and nuclei. (**d**–**f**) Quantitative mean fluorescence intensity (MFI) analysis corresponding to (**d**) SOX9, (**e**) ACAN, and (**f**) COL2 Data are presented as mean ± SD (*n* = 5; ** *p* < 0.01, **** *p* < 0.0001, ns: no significance).

To verify the synthesis of chondrogenic proteins at the translational level, immunofluorescence staining was performed (Figure 7a–c). Consistent with the RT-qPCR results, BMSCs in the SFHD-BT@PDA + US group showed extensive positive staining for the main chondrogenic transcription factor SOX9, as well as Aggrecan and Type II Collagen. Quantitative analysis of the corresponding protein immunofluorescence (Figure 7d–f) demonstrated that the expression of chondrocyte marker proteins in the piezoelectric stimulation group was significantly elevated compared with the control and unstimulated groups. All the above results indicate that the SFHD-BT@PDA hydrogel can induce the differentiation of BMSCs into chondrocytes under ultrasound stimulation.

### 2.7. Ultrasound-Stimulated SFHD-BT@PDA Hydrogel Alleviates Early OA Progression

**Figure 8:**
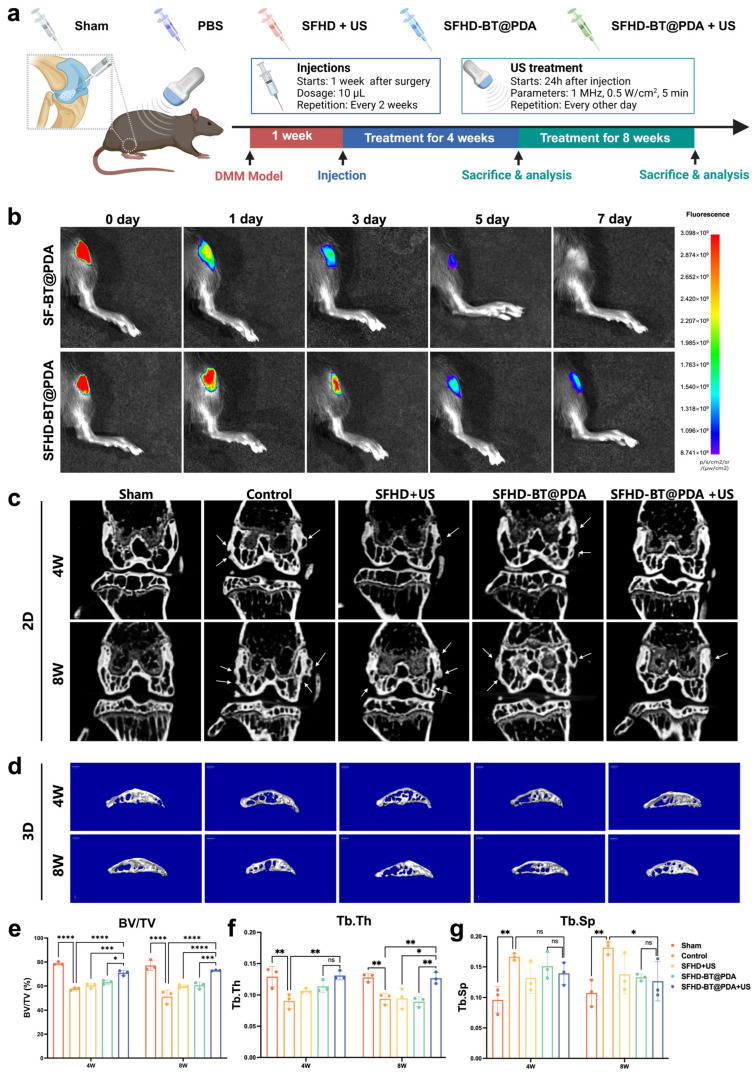
In vivo retention and therapeutic effect in the DMM-induced OA mouse model. (**a**) Schematic diagram of the animal experiment. Created in BioRender. Bigbone, B. (2026) https://BioRender.com/nngwdhi (accessed on 1 June 2026). (**b**) In vivo fluorescence imaging (IVIS) of mouse knee joints for 7 days after injection of Cy5-labeled SF-BT@PDA (non-adhesive) and SFHD-BT@PDA (adhesive) hydrogels. (**c**) Representative 2D coronal Micro-CT images of knee joints at 4 and 8 weeks post-surgery. White arrows indicate osteophytes. (**d**) 3D reconstruction images of the tibial subchondral bone. (**e**–**g**) Quantitative morphometric analysis of subchondral bone parameters: (**e**) Bone volume fraction (BV/TV), (**f**) trabecular thickness (Tb.Th), and (**g**) trabecular separation (Tb.Sp). Data are presented as mean ± SD (in all datasets, *n* = 3; * *p* < 0.05, ** *p* < 0.01, *** *p* < 0.001, **** *p* < 0.0001, ns: no significance).

To verify the therapeutic effect of the SFHD-BT@PDA hydrogel piezoelectric system on early-stage OA, we established an early-OA mouse model using the DMM method. Mice were divided into five groups: Sham (incision only, PBS injection), control (DMM + PBS), SFHD + US (DMM + SFHD hydrogel + ultrasound), SFHD-BT@PDA (DMM + composite hydrogel, no ultrasound), and SFHD-BT@PDA + US (DMM + composite hydrogel + ultrasound). One-week post-surgery, after confirming wound healing, hydrogel precursors were injected intra-articularly. For groups involving ultrasound stimulation, treatment was administered every other day starting 24 h after injection to construct an in situ piezoelectric microenvironment. Tissue samples were collected for subsequent analysis at 4 and 8 weeks post-treatment (Figure 8a).

In the dynamic and fluid-rich articular environment, long-term hydrogel retention is crucial for sustained therapeutic effects. We used an IVIS system to monitor the fluorescence of Cy5-labeled BT@PDA nanoparticles for evaluating the efficacy of the HADA adhesion component. As shown in Figure 8b, after injection (day 0), there were strong red fluorescent signals in the knees, which means that the material retention and localized delivery were achieved. Significant differences in signal attenuation were observed over time. Compared with the SF-BT@PDA group (lack of adhesive HADA component), the fluorescence signal decay in the SFHD-BT@PDA group was significantly slower. The prolonged retention time of this drug is attributed to the strong adhesion between HADA and the cartilage surface, which can resist the mechanical clearance in the articular cavity and ensure the long-term in situ effect of nanoparticles.

OA disease features include cartilage degeneration, subchondral bone sclerosis, cystic degeneration and osteophyte formation. To evaluate the therapeutic effect of piezoelectric hydrogel in maintaining joint morphology and microstructure, Micro-CT scanning was performed on the joints of mice. As shown in the 2D coronal section (Figure 8c), the Sham group showed a smooth articular surface and complete subchondral bone structure. In sharp contrast, the control group showed severe OA characteristics at 4 and 8 weeks, including rough articular surface, subchondral bone sclerosis and obvious osteophyte hyperplasia at the joint edge (shown by the white arrow), which confirmed the successful establishment of the DMM-induced OA model and the progressive characteristics of the disease. The SFHD-BT@PDA + US group demonstrated the most significant structural preservation. The joint surface remained smooth, and osteophyte formation was significantly inhibited. 3D reconstruction of the subchondral bone (Figure 8d) further confirmed these findings. The control group showed a highly porous and eroded trabecular microstructure. Conversely, the SFHD-BT@PDA + US group presented a dense and well-connected trabecular network. Quantitative analysis of trabecular parameters (Figure 8e–g) showed that SFHD-BT@PDA + US treatment significantly reversed bone loss, restoring bone volume fraction (BV/TV) to near-normal levels at 8 weeks. Consistent with this, trabecular thickness (Tb.Th) was higher and trabecular separation (Tb.Sp) was significantly lower in the treatment group compared to the control. In summary, these Micro-CT results provide strong evidence that the SFHD-BT@PDA + US system effectively mitigates pathological remodeling of subchondral bone and retards OA progression.

### 2.8. Ultrasound-Stimulated SFHD-BT@PDA Hydrogel Contributes to Cartilage Matrix Maintenance in OA

**Figure 9:**
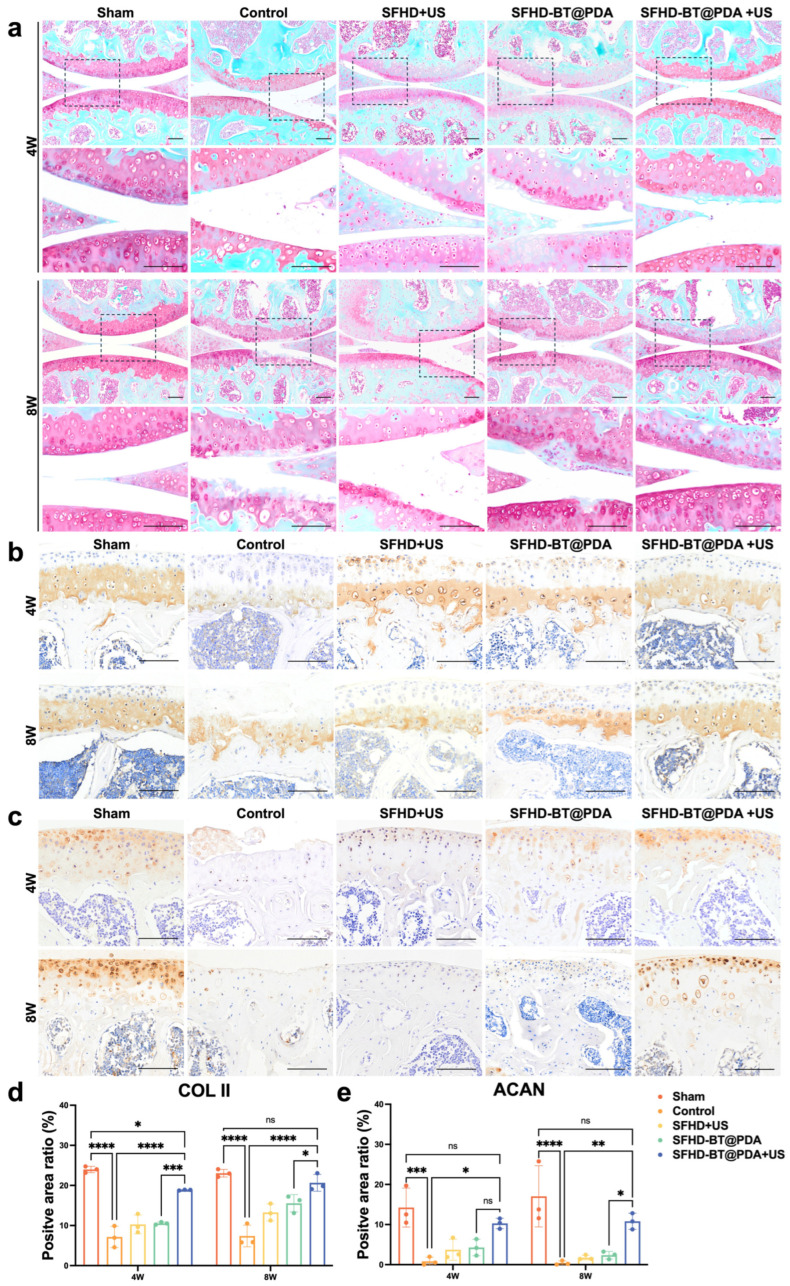
Histological evaluation of cartilage repair. (**a**) Safranin O-Fast Green staining of knee joint sections at 4 and 8 weeks. Red indicates proteoglycan content; green indicates bone/cytoplasm. The lower panel is an enlarged view of the area framed by the black dashed box in the upper panel (scale bar = 100 μm). (**b**,**c**) Immunohistochemical (IHC) staining of (**b**) Type II Collagen (COL II) and (**c**) Aggrecan (ACAN) in articular cartilage at 4 and 8 weeks. Brown staining indicates positive expression (Scale bar = 50 μm). (**d**,**e**) Quantitative analysis of the positive area ratios of (**d**) COL II and (**e**) ACAN in articular cartilage at 4 and 8 weeks. Data are presented as mean ± SD (in all datasets, *n* = 3; * *p* < 0.05, ** *p* < 0.01, *** *p* < 0.001, **** *p* < 0.0001, ns: no significance).

To evaluate the therapeutic effect of the piezoelectric hydrogel on cartilage integrity and matrix composition at the microscopic level, histological and immunohistochemical analyses were performed at 4 and 8 weeks post-treatment. Safranin O-Fast Green staining was used to observe the distribution of GAGs and the morphological structure of articular cartilage (Figure 9a). The Sham group exhibited a smooth, intact cartilage surface accompanied by uniform, intense red staining, indicating healthy proteoglycan content. The control group showed typical pathological characteristics of OA. At 4 weeks, loss of superficial proteoglycans was visible. By 8 weeks, the injury was further aggravated, the red staining in the cartilage matrix almost disappeared, and the subchondral bone was directly exposed. The SFHD + US group showed slight therapeutic effects, but cartilage surface defects remained obvious. Compared with the control group, the SFHD-BT@PDA group retained a part of the cartilage structure, but the injury site mainly exhibited fibrocartilage repair tissue, with light and uneven red staining, suggesting insufficient matrix synthesis, presenting as a typical degeneration of osteoarthritis in the early stage. However, the SFHD-BT@PDA + US group showed the most significant cartilage protection effect. Even in the 8th week, the joint surface remained smooth and continuous. The cartilage layer showed dark and uniform red staining, similar to the Sham operation group, which confirmed that piezoelectric stimulation effectively inhibited the loss of proteoglycan and promoted the retention of matrix.

We also evaluated the expression of Type II collagen and aggrecan, biomarkers related to cartilage matrix synthesis, by immunohistochemical staining to determine the biological effect of SFHD-BT@PDA + US in vivo. (Figure 9b,c). Compared with the control group, the SF + US and SFHD-BT@PDA groups showed some retention of positive signals at 4 weeks post-surgery. However, after 8 weeks, there was no obvious difference from the control group, as all showed a severe lack of positive staining, indicating massive loss of COL II and aggrecan in the extracellular matrix. Interestingly, the SFHD-BT@PDA + US group still exhibited deep and uniform positive signals after 8 weeks. This preservation of high-density COL II and aggrecan suggests that in situ electrical stimulation promoted the synthesis of hyaline cartilage-specific proteins, resulting in a therapeutic effect significantly higher than that of other groups.

Subsequently, we quantitatively analyzed the positive area percentage of these two indicators to further verify the above morphological observation (Figure 9d,e). Type II collagen and aggrecan expression levels in the SFHD-BT@PDA + US group were markedly higher than in all other treatment groups at 4 and 8 weeks. Especially at 8 weeks, the positive rate of Type II collagen in the SFHD-BT@PDA + US group was maintained at about 20%, while the expression level of aggrecan in the SFHD-BT@PDA + US group was close to that in the Sham group. Remarkably, statistical comparisons revealed that the US-driven piezoelectric stimulation (SFHD-BT@PDA + US) significantly upregulated the deposition of COL II (at 4W and 8W) and ACAN (at 8W) compared to the hydrogel alone without US (SFHD-BT@PDA). The maintenance of this high-level functional protein proves that ultrasound-triggered piezoelectric stimulation can simulate the physiological and electrical environment, effectively activate the anabolism of chondrocytes, and inhibit the matrix loss in the process of OA.

The combined results indicate that the SFHD-BT@PDA group under ultrasound stimulation can effectively inhibit osteophyte formation, prevent bone loss, mitigate pathological remodeling of subchondral bone, and simultaneously promote the secretion of cartilage-related ECM by cells, thereby achieving effective reversal of the OA disease process.

The restoration of the bioelectric microenvironment shows a promising frontier direction for preventing the pathological progress of early loss of piezoelectric properties of OA cartilage and insufficient endogenous repair ability. However, current treatment strategies are often faced with failure to reproduce the inherent “electromechanical coupling” characteristics of cartilage, or failure to adhere closely to the dynamic joint surface, resulting in insufficient in situ action time. In this study, we designed a nanocomposite hydrogel (SFHD-BT@PDA) to solve these two defects. The hydrogel combines ultrasonic piezoelectric properties with strong wet adhesion properties. By successfully simulating the physiological signals of the cartilage microenvironment, our system not only promoted the recruitment and differentiation of endogenous BMSCs in vitro, but also effectively prevented joint degeneration and subchondral bone remodeling in the mouse DMM model.

The articular cavity is a complex environment filled with synovial fluid and subjected to dynamic shear force [37]. Type II collagen, the main component of the articular cartilage surface, is electrically neutral or slightly negative under physiological conditions and the surface is highly hydrated. It is difficult for traditional hydrogels to achieve long-term retention through simple physical adsorption, and the treatment effect is often poor due to contact interface slip. To overcome this problem, we adopted a “mussel-like” strategy and introduced HADA as a molecular bridge. Macroscopic experiment confirmed SFHD-BT@PDA IVIS in vivo imaging and further confirmed its long-term retention ability in the articular cavity. At the same time, we also revealed the mechanism of this wet adhesion from the atomic scale through MD simulation. The simulation results show that HADA molecules are not randomly adsorbed, but embedded into the collagen helical groove through conformational adjustment, showing high geometric complementarity. The thermodynamic spontaneity of this process was confirmed by free-energy calculation, in which van der Waals force played a leading role and electrostatic interaction assisted localization. In particular, a stable cationic–π interaction was formed between the benzene ring of the dopamine side chain and arginine on the surface of collagen. This non-covalent interaction has a special stability in an aqueous environment. Combined with a wide range of hydrogen bond networks, the hydrogel is endowed with the ability to resist joint fluid erosion and mechanical shear, which provides a basis for subsequent continuous treatment.

Reconstructing the bioelectric microenvironment constitutes a central tenet of our therapeutic strategy. BT has good piezoelectric properties, but raw nanoparticles aggregate in solution because of their high surface energy. This uneven dispersion will not only result in hydrogel network structural damage, but also produce a very uneven spatial electric field distribution under the action of ultrasound. The uneven distribution of the local electric field may create regions of high potential, causing ROS-mediated cell damage [38]; however, in other regions, it is difficult to effectively activate the signal pathway due to the lack of potential. This heterogeneity will seriously affect the overall uniformity and safety of treatment. For this purpose, we constructed the BT@PDA core–shell structure [39]. The structure improves the dispersion of particles, makes the distribution of the electric field more uniform, and ensures the stable and consistent transmission of piezoelectric signals in the hydrogel. This design not only avoids the potential cytotoxicity, but also achieves a better biological regulation effect.

For endogenous stem cells to be effectively recruited to the inflammatory site is the prerequisite for in situ chondrocyte regeneration. This study found that under ultrasonic stimulation, the SFHD-BT@PDA group significantly improved the migration efficiency of BMSCs and made the cells directionally infiltrate the inflammatory site deep in the hydrogel. Electrotaxis supports this phenomenon: the charge on the cell membrane surface can form a dynamic potential difference in the hydrogel matrix under ultrasonic vibration, and then the cytoskeleton is polarized, thereby driving the cells to migrate directionally along the electric field gradient [10].

Regarding differentiation, our data revealed a marked upregulation of chondrogenic genes (*Sox9*, *Col2a1*, *Acan*) under piezoelectric stimulation. Notably, the concomitant rise in *Tgfb1* transcript levels offers a critical mechanistic insight. Previous studies have established that piezoelectric microcurrents can trigger Ca^2+^ influx via voltage-gated calcium channels (VGCCs), subsequently activating calcineurin-dependent pathways to potentiate TGF-β1 autocrine signaling [40,41,42]. We verify the results. The SFHD-BT@PDA in situ electrical signal may activate the TGF-β1 cascade via the classic pathway, thus driving chondrogenic transcription. Immunofluorescence confirms this at the protein level. The deposition of amounts of Type II collagen and proteoglycans confirms that the regenerated tissue presents a hyaline cartilage phenotype. Taken together, these chondrogenic differentiation and matrix-deposition findings are consistent with the recent work of Lin et al., who reported that ultrasound-activated PDA-modified BaTiO_3_-loaded hydrogels enhanced chondrogenic differentiation of BMSCs [28]. Together with that study, our results further support the concept that ultrasound-triggered piezoelectric hydrogels can provide effective electromechanical cues for cartilage matrix regeneration. More importantly, our study extends this strategy by introducing a mussel-inspired HADA/SF adhesive matrix, which enables stable wet-cartilage anchoring and improved intra-articular retention, thereby addressing a key translational challenge of injectable piezoelectric hydrogels for OA therapy.

The OA course evaluation of the comprehensive intervention effect on osteoarthritis is related to subchondral bone remodeling and osteophyte formation. Our study shows that in the DMM mouse model, simple material injection without ultrasonic intervention can provide a certain physical barrier, but the repair effect is limited in the long-term observation, and the generated tissue is mostly fibrocartilage. In contrast, the treatment group stimulated by ultrasound not only has high-quality cartilage repair but also improves the subchondral bone microstructure and restores bone volume fraction and trabecular thickness. This indicates that the piezoelectric hydrogel can prevent the progression of OA.

Although this study confirmed the potential of the SFHD-BT@PDA system in the treatment of early OA, there are still some limitations. This study only verified the curative effect in a mouse model. Considering that the size and mechanical load of human joints are much larger than those of mice, it is necessary to further evaluate its load-bearing capacity and degradation kinetics in large animal models in the future. Secondly, the pathological process of the DMM model is different from that of human primary and age-related OA. In the future, its efficacy needs to be verified in a model closer to clinical practice. At the same time, although we observed the upregulation of TGF-β1, the specific molecular mechanism by which piezoelectric signals are converted into biochemical signals still needs to be further explored. Although this ultrasound-driven piezoelectric hydrogel system shows great promise for cartilage repair, several critical challenges must be addressed before future clinical translation. On one hand, technical and administrative protocols require substantial optimization for human scale. While LIPUS seamlessly penetrates the joint capsule of small rodents, human joints (such as the knee or hip) possess significantly thicker overlying soft tissues that can attenuate acoustic energy, potentially weakening the in situ piezoelectric microcurrent generation. Optimizing ultrasound parameters or designing guided acoustic windows will therefore be essential. Furthermore, for chronic degenerative diseases like osteoarthritis, a single hydrogel injection might not cover the entire therapeutic window. This necessitates further investigation into the safety and frequency of repeated intra-articular administrations, as well as their long-term impact on joint homeostasis and user compliance.

On the other hand, the long-term biological performance and structural durability of the biomaterial demand rigorous, long-term validation. The in vivo degradation kinetics of the hydrogel matrix must be precisely synchronized with the pace of endogenous cartilage ECM deposition; premature degradation could lead to structural collapse and early loss of the embedded nanoparticles, whereas delayed degradation might physically obstruct tissue remodeling. Although the current rodent model provides valuable proof-of-concept data, it fails to replicate the demanding weight-bearing conditions and complex joint anatomy of humans. Consequently, future evaluations in large animal models—such as rabbits, sheep, or pigs—are indispensable to fully validate the structural stability, systemic biosafety, and therapeutic efficacy of this piezoelectric system under physiological load-bearing conditions.

## 3. Conclusions

This study proposes and verifies a scheme for physical microenvironment regulation and intra-articular drug retention for the treatment of early OA. At the material design level, an SFHD-BT@PDA nanocomposite hydrogel is prepared. This design introduces HADA molecular bridges, uses cation–π interaction and a geometric complementarity mechanism, overcomes the wet and sticky barrier of the articular cartilage surface, and constructs long-term material retention in the joint cavity. At the same time, the core–shell structure BT@PDA is designed to eliminate electric field distortion and convert in vitro ultrasound into uniform and safe endogenous bioelectric signals.

The prepared SFHD-BT@PDA hydrogel has a uniform porous structure, good injectability, anti-swelling property, and a degradation rate matching the cartilage repair cycle. In vitro experiments show that SFHD-BT@PDA has good biocompatibility. Under ultrasonic stimulation, the system exhibits significant galvanotaxis, recruiting BMSCs, while also effectively promoting their chondrogenic differentiation. Molecular mechanism analysis showed that the in situ piezoelectric microenvironment constructed by SFHD-BT@PDA may promote the biosynthesis of Type II collagen and GAGs by activating the TGF-β1 signaling pathway and upregulating the expression of key transcription factors such as SOX9. To conduct a more rigorous verification of these research conclusions, we evaluated the therapeutic potential of this system in the DMM model. Micro-CT analysis and histological staining showed that SFHD-BT@PDA exhibited long-term tissue retention capacity, which could inhibit osteophyte formation, protect the subchondral bone microstructure, maintain the hyaline cartilage matrix, and block the pathological progression of OA.

This study provides a new acoustic–electrical–biological coupling material for the minimally invasive intervention of early OA, provides a new idea for using the physical microenvironment to control the fate of stem cells, and also provides an innovative strategy for the future research of cartilage regeneration by combining organoid technology with a dynamic hydrogel system, showing far-reaching potential applications [43]. Future integration of this system with drug targeting technologies could enable more advanced in situ precision therapy [44].

## 4. Materials and Methods

### 4.1. Materials

BT nanoparticles, dopamine hydrochloride and HRP were purchased from Macklin (Shanghai, China). Hydrogen peroxide (H_2_O_2_), lithium bromide (LiBr) and sodium carbonate (Na_2_CO_3_) were purchased from Aladdin (Shanghai, China). Sodium hyaluronate was purchased from Yuanye Biology (Shanghai, China).

### 4.2. Preparation of BT@PDA

The preparation of dopamine-modified barium titanate nanoparticles (BT@PDA) was accomplished via a two-step method: surface hydroxylation pretreatment followed by in situ dopamine polymerization. First, to improve surface reactivity, BT was subjected to hydroxylation; 10 g of raw BT was dispersed in 200 mL of hydrogen peroxide (H_2_O_2_) solution. The reaction was conducted at 96 °C for 8 h. Post-reaction, the product was filtered, vacuum-dried at 60 °C for 12 h, and surface-hydroxylated barium titanate was obtained (BT-OH). Subsequently, the prepared BT-OH powder and dopamine hydrochloride were added together into 500 mL of alkaline Tris-HCl buffer (10 mM). Stirring of the mixture was performed at room temperature for 24 h. After that, the mixture was filtered, repeatedly washed with double distilled water and centrifuged, and repeated three times to remove DMSO, residual dopamine hydrochloride and impurities. Finally, the BT@PDA particles were collected after drying at room temperature.

Ascanning transmission electron microscope (STEM) (JEM-F200, JEOL, Akishima, Japan), acceleration voltage = 200 kV, probe current = 7.475 nA) was used to observe the morphology of BT@PDA nanoparticles, and the elemental composition of BT@PDA nanoparticles was analyzed by EDS element surface scanning. The particle size of B and zeta potential were measured by a dynamic light scattering analyzer (Zetasizer Pro, Malvern Panalytical, Malvern, UK). Before measurement, the BT and BT@PDA nanoparticles were uniformly dispersed in 1× Phosphate-Buffered Saline (1× PBS, pH 7.4, ionic strength ≈ 0.15 M) and evaluated at room temperature. Using an X-ray diffractometer (XRD, Germany D8 Venture, Bruker, Karlsruhe, Germany) in the scanning range of 10° to 70°, for BT and BT@PDA, the crystal structure of nanoparticles was characterized and analyzed. For BT and BT@PDA, after 1 h of polarization at 2.04 kv voltage, the d33 test was carried out by the withstand voltage tester (rk2671a, Merrick, Shenzhen, China), and the piezoelectric response was measured by the contact mode of the piezoelectric response force microscope (PFM, Bruker, Karlsruhe, Germany).

### 4.3. Preparation of HADA

The preparation of the HADA adhesive component utilized a modified EDC/NHS activation method [45]. Briefly, sodium hyaluronate (Mw = 100 kDa, 100 mg) was dissolved in PBS buffer (pH 5.5) to prepare a 1% (*w*/*v*) solution. In order to improve activation efficiency, EDC (69 mg, 0.36 mmol) and NHS (41.4 mg, 0.36 mmol) were pre-dissolved in a small amount of dimethyl sulfoxide (DMSO) and then added dropwise to the HA solution, stirring for 30 min for activation. Next, dopamine hydrochloride (56.9 mg, 0.30 mmol) dissolved in DMSO was added to the reaction system. The volume ratio of DMSO to the aqueous phase was controlled to be less than 20% to prevent HA precipitation. To prevent self-oxidative polymerization of dopamine under alkaline or aerobic conditions, the pH was strictly maintained at 5.5, and the reaction was conducted under nitrogen protection in the dark. The reaction was stirred at room temperature for 12 h. After that, the mixture was loaded into a dialysis bag (MWCO = 7000 Da) and dialyzed against acidic deionized water (pH 5.0, containing trace hydrochloric acid) for 2 days to remove DMSO, unreacted dopamine, and byproducts, and to inhibit oxidation; subsequently, it was dialyzed in deionized water for 1 day to remove salts. The purified solution was pre-frozen and lyophilized at −80 °C for 72 h to obtain a white flocculent HADA product, which was stored in a desiccator at −20 °C.

The chemical structure of HADA (1% solution) was analyzed by ^1^H NMR spectroscopy (Bruker AVANCE 500 MHz, Bruker BioSpin, Fällanden, Switzerland), Fourier transform infrared spectroscopy (FTIR, TENSOR 27, Bruker Optics, Ettlingen, Germany), and UV-Vis absorption spectroscopy (UV-3600 plus, Shimadzu, Kyoto, Japan). The detection wavenumber range for FTIR was 4000–400 cm^−1^, and the wavelength range for UV-Vis was 230–350 nm.

### 4.4. Preparation of SF

The SF was prepared using the classic degumming–dissolution–dialysis method [46,47,48]. First, 5 g of dry *Bombyx mori* cocoons were weighed, sectioned into pieces, and soaked in 500 mL of boiling 0.02 mol/L sodium carbonate (Na_2_CO_3_) solution for 30 min to remove sericin. After removing the silk, it was rinsed repeatedly with deionized water until the pH of the wash solution was near neutral (pH ≈ 7), and the degummed fibers were dried. Next, 2 g of dry silk fibers were accurately weighed and dissolved in 20 mL of 9.3 mol/L lithium bromide (LiBr) solution. The mixture was stirred continuously in a water bath at 60 °C for 4 h until the fibers were completely dissolved, obtaining a transparent, brownish-yellow viscous liquid. The solution was placed into a dialysis bag (MWCO = 3500 Da) and dialyzed against 5 L of deionized water for 72 h to remove salts, with the water changed every 8 h. After dialysis, the solution was centrifuged at 9000 rpm at 4 °C for 20 min to remove insoluble impurities. The supernatant was collected, and the final concentration was determined and adjusted to 5% (*w*/*v*) by weighing and drying method. The prepared SF solution has a transparent appearance and no precipitation, and is stored at 4 °C for standby.

### 4.5. Preparation of SFHD-BT@PDA Hydrogel

The SFHD-BT@PDA composite hydrogel was prepared by HRP/H_2_O_2_ mediated enzymatic crosslinking method. First, the HADA conjugate was dissolved in a 5% (*w*/*v*) silk fibroin (SF) solution to prepare an SFHD precursor solution to prepare a 1% HADA mass fraction SFHD precursor solution. Then, BT@PDA nanoparticles were added to the solution and fully mixed, with the final nanoparticle concentration adjusted to 1%. To construct the hydrogel network, crosslinking initiators were introduced into the mixture. Horseradish peroxidase (HRP, 0.5 U/mL) and then H_2_O_2_ (0.05% *w*/*v*) were added to initiate the crosslinking reaction. The mixture was stirred rapidly to ensure uniformity, and then left to stand until the sol–gel transition was completed to obtain the SFHD-BT@PDA hydrogel.

In addition, to verify the effect of PDA modification on the spatial dispersion of BT nanoparticles in the SFHD hydrogel matrix, the nanoparticles were fluorescently labeled with DiD dye. Briefly, the prepared BT@PDA nanoparticles were dispersed in diluted DiD solution (5 mg/mL) and dark-incubated for 30 min to achieve effective physical adsorption. After incubation, unbound free dyes were completely removed by repeated centrifugation and washing with PBS three times. The fluorescent-labeled BT@PDA nanoparticles were added to the precursor solution of SFHD to prepare composite hydrogels. After gelation, CLSM (FV3000, Olympus, Hachioji, Tokyo, Japan) was used for imaging the internal microstructure of the hydrogel at the excitation wavelength of 644 nm.

### 4.6. Characterization of SFHD-BT@PDA Composite Hydrogel

#### 4.6.1. Morphological Characterization

To observe the morphology of hydrogel with different components, the hydrogel samples were frozen in liquid nitrogen for 30 min and then lyophilized. The dried hydrogel surface was coated by gold-sputtering, and then observed by the scanning electron microscope (SEM, GeminiSEM 300, Zeiss, Oberkochen, Germany, 10 kV). The elemental composition of the SFHD-BT@PDA hydrogel was analyzed by EDS elemental mapping.

#### 4.6.2. Injectability Evaluation

The macroscopic injectability of the hydrogel was evaluated to simulate the in situ gelation process for minimally invasive administration. Briefly, the hydrogel precursor solution was prepared, followed by the sequential addition of horseradish peroxidase (HRP, 0.5 U/mL) and hydrogen peroxide (H_2_O_2_, 0.05% *w*/*v*) to initiate the crosslinking reaction. The reacting mixture was immediately loaded into a standard commercial syringe and manually extruded through a 26-gauge (26G) needle at room temperature to write predefined macroscopic patterns. The sol–gel transition was completed within 5 min post-extrusion.

#### 4.6.3. Tissue Adhesion Strength Measurement

The tissue adhesive properties of the SFHD-BT@PDA hydrogels were evaluated using a standard lap-shear test according to the ASTM F2255 standard. Briefly, fresh porcine cartilage was cut into uniform pieces (10 mm × 10 mm) and firmly attached to the ends of two separate acrylic plates. The hydrogel precursor (10 mm × 10 mm × 3 mm) was then sandwiched between the two cartilage surfaces. To ensure uniform contact and robust interfacial bonding during in situ gelation, a constant pre-load force of 1 N was applied to the overlapped area for a pre-contact time of 5 min at room temperature. Following gelation, one side of the sample was fixed, and a tensile force was applied to the other side by a universal material testing machine (Instron 5943B, Instron, Norwood, USA) at a constant crosshead speed of 10 mm/min until failure.

#### 4.6.4. Piezoelectric and Ultrasound-Triggered Current Measurement

For the assessment of hydrogel piezoelectric properties, an in vitro ultrasound–piezoelectric signal acquisition platform was established. Cylindrical hydrogels (10 mm diameter, 5 mm thickness) were sandwiched between two copper foil electrodes and horizontally secured using a metal stand. To prevent short circuits or electrical interference from direct contact between the ultrasound probe and the electrodes, insulating tape was pre-attached to the targeted testing area on the upper copper plate. A pulsed-ultrasound probe was positioned vertically in tight contact with the insulated region, and the electrodes were connected to a digital oscilloscope via testing clips (Figure S4).

The evaluation consisted of two parts. First, to assess the baseline and material-specific piezoelectric responses, the real-time open-circuit voltage was recorded for three groups: a Blank group (SFHD-BT@PDA hydrogel without ultrasound stimulation), an SFHD group (pure SFHD hydrogel with ultrasound stimulation), and an SFHD-BT@PDA group (composite hydrogel with ultrasound stimulation). Second, to investigate the frequency-dependent mechano-electrical conversion, ultrasonic stimulation was applied to the SFHD-BT@PDA hydrogels at frequencies of 1 MHz and 3 MHz (both at a power density of 0.5 W/cm^2^).

#### 4.6.5. Rheological Assessment

A rheometer (DHR-20, TA Instruments, New Castle, DE, USA) was used to evaluate the rheology of the hydrogels. Dynamic frequency scanning from 0.1 to 100 rad s^−1^ was performed at 1% strain.

#### 4.6.6. Mechanical Characterization

The mechanical properties of the hydrogels were evaluated using a universal materials testing machine (CMT5504, SANS, Shenzhen, China) at room temperature. Cylindrical hydrogel samples (10 mm × 10 mm × 3 mm) were prepared for all unconfined mechanical tests.

Compressive Test: Unconfined compression was performed at a constant crosshead speed of 0.5 mm/min until reaching a maximum strain of 80%. The compressive modulus was calculated from the slope of the initial linear elastic region (0–10% strain) of the resulting stress–strain curve.

Cyclic Loading Test: To assess dynamic fatigue resistance and shape recoverability, the hydrogel samples were subjected to 10 consecutive compression–unloading cycles at a constant strain of 50% with a consistent loading rate, without resting intervals between cycles.

Stress–Relaxation Test: The hydrogels were rapidly compressed to a predefined strain of 50% and held constant. The stress decay was continuously recorded over a holding period of 300 s to characterize the viscoelastic energy dissipation behavior of the crosslinked network.

#### 4.6.7. Swelling Ratio Test

Hydrogel swelling performance was characterized by gravimetric analysis. First, the lyophilized hydrogel was weighed to record its initial dry weight ($W_{d}$). At preset time points, take out the sample and gently dry the excess surface water with filter paper before weighing its wet weight ($W_{s}$). This process was repeated until the hydrogel reached swelling equilibrium. The swelling ratio was calculated as follows:

(1) $$Swelling\;Ratio\left(\%\right)=\frac{W_{s}-W_{d}}{W_{d}}\times 100\%$$

#### 4.6.8. Degradation Performance Evaluation

The initial weight ($W_{0}$) of the lyophilized hydrogel was determined, and then the sample was immersed in the protease XIV solution prepared with 10 mmol/L PBS (enzyme activity 5 u/mL) and incubated at 37 °C with constant temperature oscillation at 100 rpm. The fresh protease XIV solution was replaced every 24 h. The hydrogel was sampled at the preset time nodes (2, 4, 6, 8, 10, 12, 14d) and the sample mass was weighed, which was recorded as ($W_{t}$). The residual mass ratio of the hydrogel is calculated by the following formula:

(2) $$Remaining\;Weight\left(\%\right)=\frac{W_{t}}{W_{0}}\times 100\%$$

### 4.7. In Vitro Cell Culture

BMSCs were utilized for in vitro validation. Primary BMSCs were maintained in complete medium consisting of α-MEM (Corning, NY, USA), 1% penicillin–streptomycin (Gibco, NY, USA), and 10% FBS (Sigma, MO, USA) at 37 °C in a 5% CO_2_ atmosphere. The medium was refreshed every two days, and cells from passages 3–5 were used for subsequent experiments.

#### 4.7.1. Cytotoxicity Assessment

The CCK-8 assay was used to evaluate the cytotoxic potential of piezoelectric hydrogels under ultrasound stimulation. SFHD-BT@PDA hydrogels loaded with varying concentrations of BT@PDA (0.25%, 0.5%, 1%, 2%, and 4%) were injected into 96-well plates, gelled, and UV-sterilized for 2 h. BMSCs were seeded onto the hydrogel surfaces (5 × 10^3^ cells/well). For ultrasound intervention groups (SFHD + US and SFHD-BT@PDA + US), stimulation was applied to the plate bottoms starting on day 2 post-seeding and repeated every 48 h (Parameters: 1 MHz, 0.5 W/cm^2^, 5 min duration). Cell viability was quantified on days 1, 3, and 7. At the pre-set time point, the culture medium was replaced with fresh medium containing 10% CCK-8 reagent (Beyotime, Shanghai, China), and cells were incubated at 37 °C for 2 h. To avoid interference, a supernatant transfer method was employed: 100 μL of the reacted supernatant was carefully transferred to a new 96-well plate, and absorbance was measured at 450 nm using a microplate reader. For live/dead staining, hydrogels were formed in 24-well plates and UV-sterilized. BMSCs were seeded on the hydrogels and cultured for 3 days. Ultrasound stimulation was applied according to the above method. Cells were stained with Calcein AM/PI double staining reagent to observe cell viability, and fluorescent images were taken by an inverted microscope (CKX3-SLP, Olympus, Hachioji, Tokyo, Japan).

#### 4.7.2. Cell Migration Assay

To simulate and observe the recruitment process of endogenous stem cells from the host tissue to the implantation site, the hydrogel-covered scratch test was used in this study. BMSCs were seeded in 24-well plates at a density of 1 × 10^5^ cells/well, and cultured for 24 h to form a cell monolayer. Then, we used a sterile 200 μL pipette tip to create a standard linear scratch. After washing with PBS to remove cell debris, we gently added 500 μL of SFHD or SFHD-BT@PDA hydrogel to cover the cell monolayer. Serum-free medium was then added to immerse the gel. US (1 MHz, 0.5 W/cm^2^, 5 min) was performed immediately after gelling and liquid addition. After 24 h, cells were stained with Calcein-AM, and fluorescent images were collected, and cell migration was observed and analyzed.

In addition, to investigate the three-dimensional growth and infiltration behavior of BMSCs on the specific hydrogel, BMSCs were seeded on the hydrogel and cultured for 7 days. The culture medium was changed every two days, and the ultrasound intervention group was stimulated every other day. At the end of culture, the cell hydrogel complex was removed and stained with Calcein-AM/PI(Beyotime, Shanghai, China). The 3D spatial distribution and infiltration depth of cells were observed by Z-stack using a CLSM.

#### 4.7.3. Cell Differentiation

Hydrogels with different components were prepared in 12-well plates and sterilized as described above. BMSCs were seeded onto the hydrogels and cultured in chondrogenic induction medium containing 1 mM sodium pyruvate (Sigma), 10 ng/mL TGF-β3 (Meilunbio, Dalian, China), 0.1 μM dexamethasone (Sigma, MO, USA), 1% ITS premix (Oricellbio, Taicang, Suzhou, China), and 1 μM ascorbate-2-phosphate (Sigma, MO, USA). Ultrasound groups received stimulation every other day.

The expression levels of chondrogenic genes (SOX9, ACAN, and Col II) were detected by qRT-PCR after 14 days of culture. Total RNA was extracted from the samples, followed by reverse transcription into cDNA. GAPDH was used as the internal reference gene for normalization. The relative mRNA expression levels of target genes were calculated using the comparative Ct method (2 − ΔΔCt), with the control group used as the calibrator.

For sulfated glycosaminoglycan (GAG) detection, cells were cultured in SFHD or SFHD-BT@PDA hydrogel extracts prepared with chondrogenic medium. After 7 and 14 days, samples were fixed with 4% paraformaldehyde and stained with Alcian Blue (1%, pH = 2.5). After staining, the cells were washed with 6 M guanidine hydrochloride (Beyotime, Shanghai, China) to elute the dye and captured by a multifunctional microplate reader.

For immunofluorescence, cells were fixed after 14 days, permeabilized with 0.1% Triton X-100 for 10 min, and blocked with 5% BSA for 1 h. Samples were incubated overnight at 4 °C with primary antibodies against Col II, SOX9, and ACAN (1:1000, Abcam, Cambridge, UK), followed by incubation with FITC-conjugated goat anti-rabbit IgG H&L secondary antibody (1:5000) for 1 h in the dark. Nuclei were counterstained with DAPI. Fluorescent signals on the hydrogel surface were captured using confocal laser scanning microscopy.

For fluorescence quantification, images of the same marker were acquired under identical confocal settings. The F-actin channel was used only to define the cell-covered region of interest (ROI), and the MFI of the target marker was measured from the independent green FITC channel within this ROI using ImageJ (v1.54t) [49]. Background-corrected MFI values were expressed as arbitrary units (a.u.) for comparison among groups.

### 4.8. In Vivo Performance Characterization

Female C57BL/6 mice (7–8 weeks old) were purchased from Changzhou Cavens Laboratory Animal Co., Ltd. (Cavens Laboratory Animal Co., Ltd., Changzhou, Jiangsu, China). The mice were maintained at 20–25 °C with 50% humidity under a 12 h light/dark cycle, with free access to water and rodent food. All animal experiments followed protocols approved by the Ethics Committee of Shanghai University (No. ECSHU 2025–198).

#### 4.8.1. Model Construction and Grouping Intervention

The C57BL/6 female mice were adapted for one week and then divided into groups. The osteoarthritis (OA) model was established using the destabilization of the medial meniscus (DMM) method. Mice were anesthetized with isoflurane (5% induction, 2% maintenance). A 2 mm incision was made on the medial side of the right knee joint to expose and sever the medial meniscotibial ligament, thereby destabilizing the joint, followed by layered suturing. One week after surgery, mice with well-healed incisions and no infection were randomly divided into 5 groups (*n* = 8 per group): Sham group (incision without ligament severance, PBS injection), control group (DMM + PBS), SFHD + US group (DMM + SFHD hydrogel + ultrasound), SFHD-BT@PDA group (DMM + composite hydrogel, no ultrasound), and SFHD-BT@PDA + US group (DMM + composite hydrogel + ultrasound).

For administration, after anesthesia, 10 μL of the corresponding reagent was slowly injected into the joint cavity from the lateral edge of the patella. The knee joint was gently rubbed for 10 s to ensure uniform distribution. The injections were repeated every two weeks. For ultrasound-treated groups (SFHD + US and SFHD-BT@PDA + US), treatment began 24 h after injection and was performed every other day (1 MHz, 0.5 W/cm^2^, 5 min).

#### 4.8.2. In Vivo Imaging and Histological Evaluation

To evaluate hydrogel retention in the joint cavity, an SF-BT@PDA group was established as a control lacking the HADA component. Cy5-labeled BT@PDA nanoparticles were dispersed in SF solution to prepare the precursor. Using the same DMM procedure, an independent cohort of OA mice was divided into two groups (SF-BT@PDA and SFHD-BT@PDA, *n* = 3 per group), and 10 μL of SF-BT@PDA solution was injected into the right knee of OA mice. The retention of nanoparticles in the SF-BT@PDA and SFHD-BT@PDA groups was monitored using an IVIS system on days 0, 1, 3, 5, and 7.

Histological evaluation was performed at weeks 4 and 8 post-treatment. Four mice per group were euthanized via cervical dislocation under deep anesthesia at each time point. Specimens of the right knee joint, heart, liver, spleen, lung, and kidney were harvested and fixed in 4% paraformaldehyde for 48 h. The harvested major organs (heart, liver, spleen, lung, and kidney) were subsequently embedded in paraffin, sectioned, and stained with Hematoxylin and Eosin (H&E) to evaluate systemic biosafety. Samples were first scanned by Micro-CT to analyze bone microstructural changes, then decalcified in 10% ethylenediaminetetraacetic acid (EDTA, pH 7.4) for 4 weeks and embedded in paraffin [50]. Subsequently, 10 μm thick sections were obtained via a microtome. Safranin O-Fast Green staining was used to observe cartilage morphology and matrix distribution. Meanwhile, immunohistochemistry was employed to evaluate the expression levels of ACAN and Type II collagen in the defect area to comprehensively assess cartilage repair. All histological evaluations and Micro-CT data analyses were performed by two independent researchers in a blinded manner.

### 4.9. Statistical Analysis

All quantitative data are expressed as the mean ± standard deviation (SD). Statistical analyses were performed using GraphPad Prism 1(v10.5.0) and OriginPro 2021 (v9.8.0.200) software. For comparisons among three or more groups, a one-way analysis of variance (ANOVA) followed by Tukey’s post hoc test was utilized. For data involving multiple variables, a two-way ANOVA was applied. A *p*-value of less than 0.05 was considered statistically significant (* *p* < 0.05, ** *p* < 0.01, *** *p* < 0.001, **** *p* < 0.0001).

## Data Availability

The original contributions presented in this study are included in the article/Supplementary Material. Further inquiries can be directed to the corresponding authors.

## Supplementary Materials

The following supporting information can be downloaded at https://www.mdpi.com/article/10.3390/gels12070630/s1: Figure S1: Characterization of the morphology and piezoelectric properties of nanoparticles; Figure S2: Schematic illustration of the experimental setup for piezoelectric coefficient (d_33_) testing; Figure S3: EDS elemental mapping of SF-BT (top) and SF-BT@PDA (bottom); Figure S4: *In vitro* ultrasound-piezoelectric signal acquisition platform; Figure S5: Time-dependent changes of structural parameters during the 100 ns molecular dynamics simulation; Figure S6: Quantitative analysis of glycosaminoglycan (GAG) deposition via Alcian Blue staining at day 7 and day 14; Figure S7: H&E staining of heart, liver, spleen, lung, and kidney tissues 8 weeks after hydrogel injection.

## Abbreviations

The following abbreviations are used in this manuscript:

OA	Osteoarthritis
SF	Silk Fibroin
HADA	Dopamine-Functionalized Hyaluronic Acid
BT@PDA	Polydopamine-Coated Barium Titanate
BT	Barium Titanate
PDA	Polydopamine
MSCs	Mesenchymal Stem Cells
SOX9	SRY-box Transcription Factor 9
TGF-β	Transforming Growth Factor-beta
DMM	Destabilization of the Medial Meniscus
US	Ultrasound
ECM	Extracellular Matrix
LIPUS	Low-Intensity Pulsed Ultrasound
MD	Molecular Dynamics
BMSCs	Bone Marrow Mesenchymal Stem Cells
STEM	Scanning Transmission Electron Microscopy
EDS	Energy-Dispersive X-ray Spectroscopy
DLS	Dynamic Light Scattering
XRD	X-ray Diffraction
PFM	Piezoelectric Force Microscopy
FTIR	Fourier Transform Infrared Spectroscopy
UV-Vis	Ultraviolet–Visible Spectroscopy
HRP	Horseradish peroxidase
CLSM	Confocal Laser Scanning Microscopy
SEM	Scanning Electron Microscopy
ACAN	Aggrecan
GAG	Glycosaminoglycan
IVIS	In Vivo Imaging System
Micro-CT	Micro-Computed Tomography
EDTA	Ethylenediaminetetraacetic Acid
H&E	Hematoxylin and Eosin
TEM	Transmission Electron Microscopy
NND	Nearest Neighbor Distance
RMSF	Root Mean Square Fluctuation
SASA	Solvent Accessible Surface Area
DMSO	Dimethyl sulfoxide
EDC/NHS	1-Ethyl-3-(3-dimethylaminopropyl)carbodiimide/N-Hydroxysuccinimide
Tb.Th	Trabecular Thickness
Tb.Sp	Trabecular Separation
