# Supplementary material for: An Ultrasound-Responsive Bio-Adhesive Piezoelectric Hydrogel for Osteoarthritis Cartilage
Source: Gels. 2026 Jul 15;12(7):630. doi: 10.3390/gels12070630 (PMC13409585; doi:10.3390/gels12070630)
Supplement: Supplementary file 1 [file gels-12-00630-s001.zip › gels-4390224-supplementary.pdf]

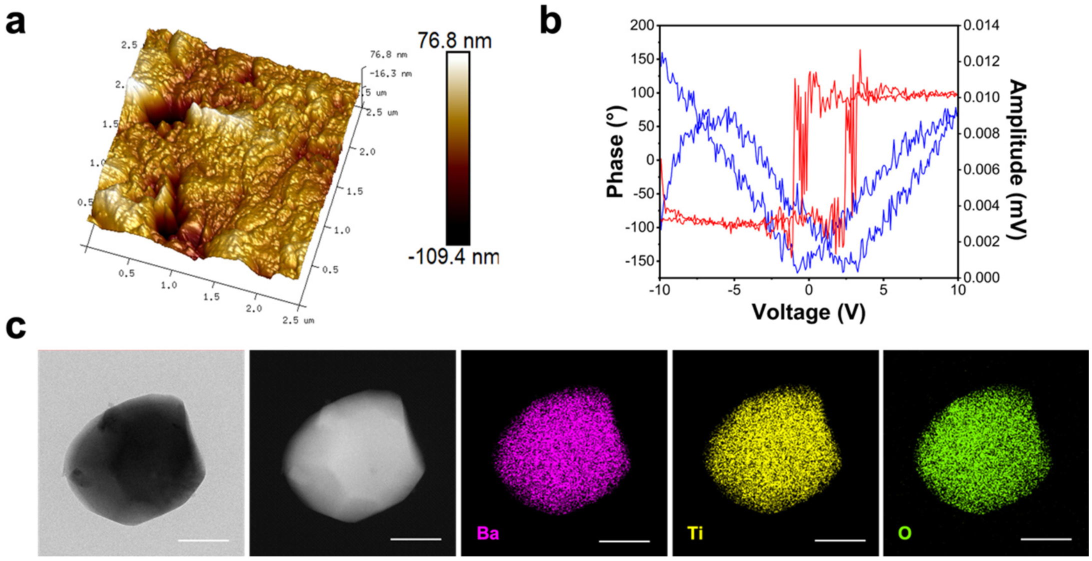

Supplementary Figure S1. Characterization of the morphology and piezoelectric properties of nanoparticles: (a) Piezoresponse force microscopy (PFM) characterization of BT nanoparticles. (b) Corresponding PFM phase hysteresis loop and amplitude butterfly loop of BT nanoparticles. **(c)** STEM image and corresponding EDS elemental mapping of Ba, Ti and O for BT nanoparticles. (Scale bar = 200 nm)

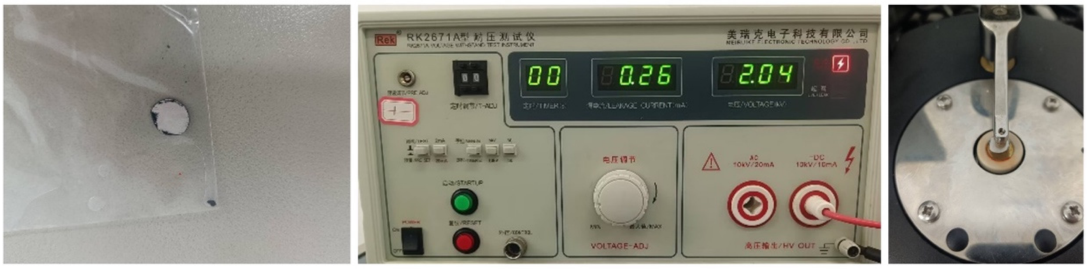

Supplementary Figure S2. Schematic illustration of the experimental setup for piezoelectric coefficient (d_{33}) testing. The three key components are sequentially presented: (left) the fabricated sample, (middle) the host measurement system, and (right) the probe for signal detection.

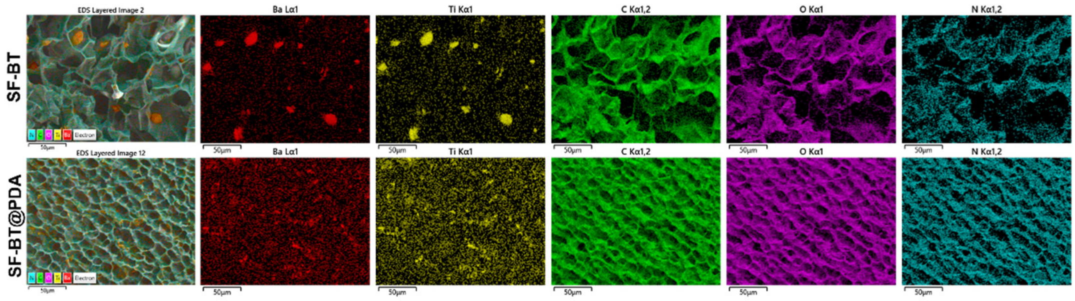

Supplementary Figure S3. EDS elemental mapping of SF-BT (top) and SF-BT@PDA (bottom).

(Scale bar = 50 μm)

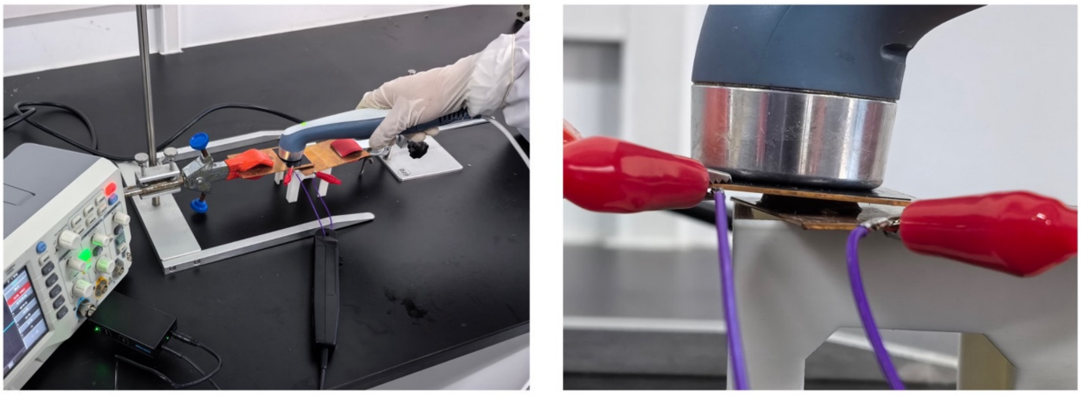

Supplementary Figure S4. *In vitro* ultrasound-piezoelectric signal acquisition platform.

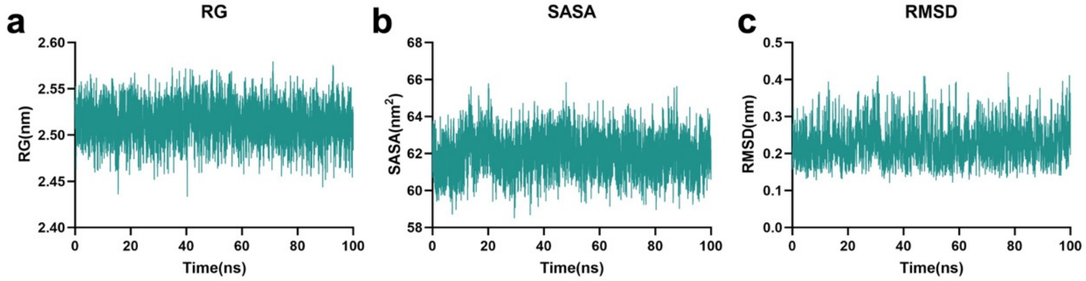

Supplementary Figure S5. Time-dependent changes of structural parameters during the 100 ns molecular dynamics simulation. Subfigures present the evolution of (a) the radius of gyration (Rg, nm), (b) the solvent accessible surface area (SASA, nm^2), and (c) the root mean square deviation (RMSD, nm). Simulations were conducted for 100 ns, with data sampled every 10 ns.

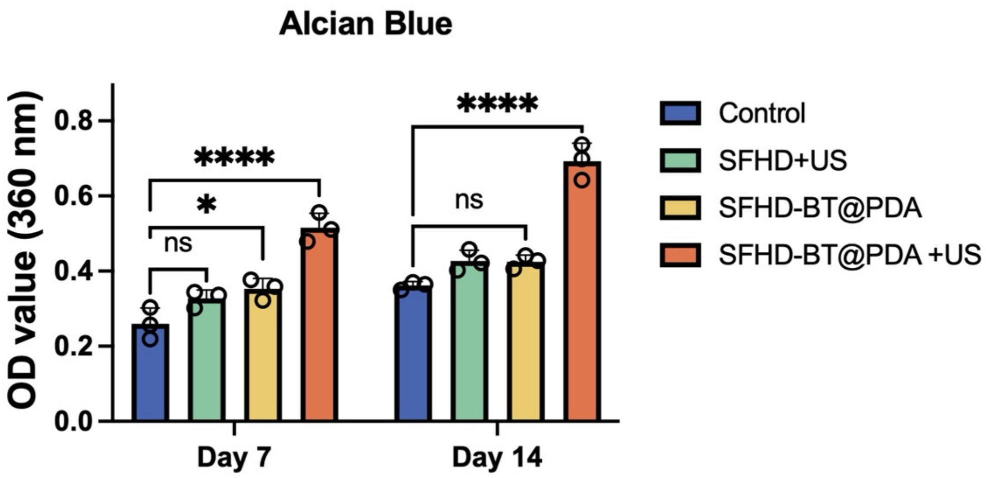

Supplementary Figure S6. Quantitative analysis of glycosaminoglycan (GAG) deposition via Alcian Blue staining at day 7 and day 14. The optical density (OD) values of the eluted dye were measured at 360 nm. Data are presented as mean ± SD (**$p < 0.01$, ***$p < 0.001$, ****$p < 0.0001$, ns: no significance).

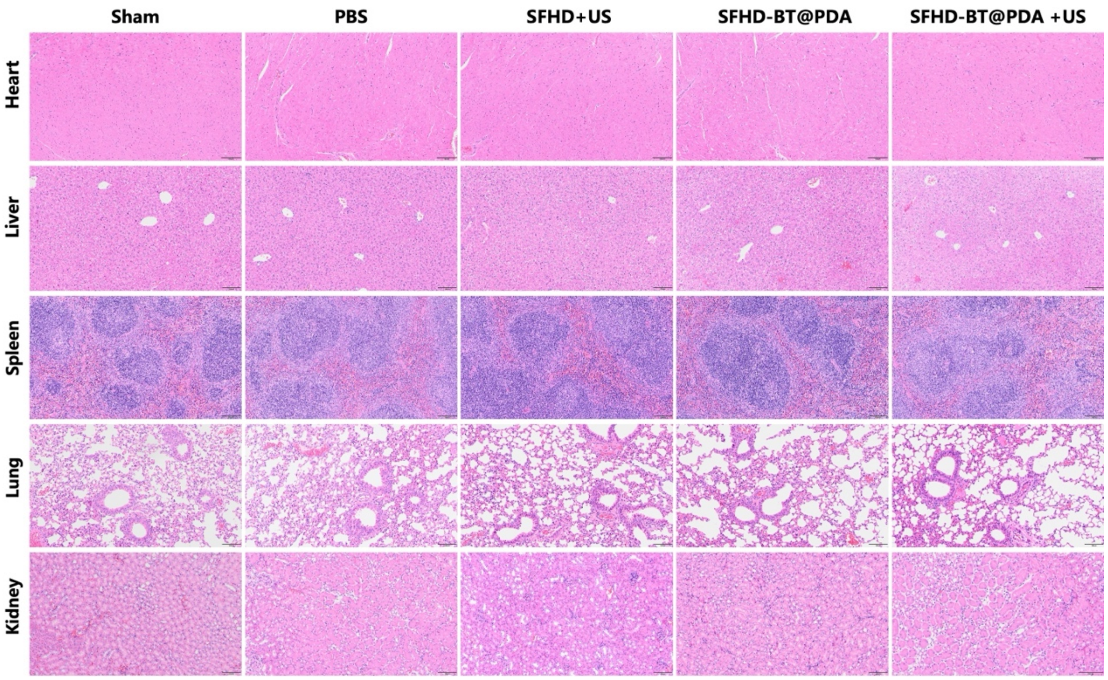

Supplementary Figure S7. H&E staining of heart, liver, spleen, lung, and kidney tissues 8 weeks after hydrogel injection (Scale bar = 100 μm).
